# Differential sensitivity to infections and antimicrobial peptide-mediated immune response in four silkworm strains with different geographical origin

**DOI:** 10.1038/s41598-017-01162-z

**Published:** 2017-04-21

**Authors:** Ottavia Romoli, Alessio Saviane, Andrea Bozzato, Paola D’Antona, Gianluca Tettamanti, Andrea Squartini, Silvia Cappellozza, Federica Sandrelli

**Affiliations:** 1 Department of Biology, University of Padova, Padova, Italy; 2 CREA - Honey Bee and Silkworm Research Unit, Padova Seat, Padova, Italy; 3 Department of Biotechnology and Life Sciences, University of Insubria, Varese, Italy; 4 Department of Agronomy, Food, Natural Resources, Animals and Environment, University of Padova, Padova, Italy

## Abstract

The domesticated silkworm *Bombyx mori* has an innate immune system, whose main effectors are the antimicrobial peptides (AMPs). Silkworm strains are commonly grouped into four geographical types (Japanese, Chinese, European and Tropical) and are generally characterised by a variable susceptibility to infections. To clarify the genetic and molecular mechanisms on which the different responses to infections are based, we exposed one silkworm strain for each geographical area to oral infections with the silkworm pathogens *Enterococcus mundtii* or *Serratia marcescens*. We detected a differential susceptibility to both bacteria, with the European strain displaying the lowest sensitivity to *E*. *mundtii* and the Indian one to *S*. *marcescens*. We found that all the strains were able to activate the AMP response against *E*. *mundtii*. However, the highest tolerance of the European strain appeared to be related to the specific composition of its AMP cocktail, containing more effective variants such as a peculiar Cecropin B6 isoform. The resistance of the Indian strain to *S*. *marcescens* seemed to be associated with its prompt capability to activate the systemic transcription of AMPs. These data suggest that *B*. *mori* strains with distinct genetic backgrounds employ different strategies to counteract bacterial infections, whose efficacy appears to be pathogen-dependent.

## Introduction

Insects are characterised by an innate immune response involving cellular and humoral components. During systemic infections, these defence mechanisms act in the hemolymph (the invertebrate blood). The cellular response is mainly driven by hemocytes responsible for pathogen phagocytosis, encapsulation and nodule formation. The humoral response includes i) the activation of the Phenoloxidase system, which triggers the synthesis of melanin and in turn contributes to the encapsulation of invading organisms, and ii) the production of several immune effectors, such as lysozyme, reactive oxygen and nitrogen species, and antimicrobial peptides (AMPs)^1–4^. The most characterised effectors are AMPs, a wide and heterogeneous group of small peptides with a variable amino acid (aa) composition and chain length^5^. During systemic infections, pathogens are recognised by a variety of Pattern Recognition Receptors (PRRs) which promote AMP synthesis in hemocytes and in the fat body, mainly *via* activation of the Toll and Immune deficiency (Imd) pathways^2, 6–9^. After proteolytic maturation, AMPs are released into the hemolymph, where they counteract invading pathogens^5, 10, 11^. In addition, several epithelia, such as those of the epidermis and gut, are able to promote AMP local synthesis, fundamental in the protection against epidermal or oral microbial exposures^12–14^.

Susceptibility to infections has been associated with genetic variability in the immune response. In *Drosophila melanogaster*, genetic polymorphisms and/or transcriptional variations in different immune components have been shown to correlate with a differential pathogen-sensitivity in both natural populations and laboratory strains^15–19^.

The domesticated silkworm *Bombyx mori* has been extensively studied as a model organism for *Lepidoptera* genetics and for its economic value in silk industry^20^. Since silkworm infectious diseases can cause crop losses of ~27–35%^21^, great attention has been posed on the study of *B*. *mori* immune mechanisms. *B*. *mori* has at least 24 AMP and 13 AMP-like genes. Among them, *moricin* (*mor*) and *lebocin* (*leb*) are present as single genes, while *attacins* (*att*), *cecropins* (*cec*), *defensins* (*def*), and *gloverins* (*glv*) have been detected as gene families composed by variable number of paralogues^22–26^. Both *att* and *def* families consist of two genes (*att1* and 2; *defA* and *B*), *glv* comprises four paralogues (*glv1*-*4*), *cec* was detected as a family of fourteen elements, classified in sub-types and including two *cecA* (*A1* and *A2*), six *cecB* (*B1*-*B6*), one *cecC*, two *cecD* (*D* and *D2*), one *cecE*, and two *enbocins* (*enb* 1 and 2)^23–26^. Furthermore, several factors homologous to *Drosophila* PRRs and multiple elements of the canonical Toll and Imd pathways have been described^2, 24^. Genome-wide transcriptional studies performed in silkworm laboratory strains revealed a general activation of all these elements after pathogen exposure^27, 28^.

*B*. *mori* domestication occurred around 5,000 years ago and currently more than 4,000 silkworm strains (including geographical, inbred and mutant strains) are available worldwide^29–31^. Geographical strains are broadly divided into four main groups: Japanese, Chinese, European and Tropical. Different observations indicate that these groups are characterised by a diverse resistance to pathogen diseases, usually inversely correlated with silk production. Silkworms from temperate regions (i.e. Europe and some regions of Japan and China) generally exhibit good silk yields but a lower resistance to infections, while tropical strains display a higher tolerance to microbial exposures but a scarce fibre production^30, 32, 33^. Other studies detected a variable sensitivity to infections in strains originating from the same geographical areas^34–36^. They also indicated that in *B*. *mori* pathogen-susceptibility is a complex phenotypic trait controlled by multiple genes^34, 35^. However, the possible molecular mechanisms governing the silkworm differential pathogen-sensitivity are still unclear.

Here we report the characterisation of the humoral immune response of four geographical silkworm strains derived from the four main regions, reared in germ-free conditions and orally exposed to the Gram-positive *Enterococcus mundtii* or the Gram-negative *Serratia marcescens* bacteria, which are the causative agents of the silkworm-specific diseases *flacherie* and *bacterial septicaemia*, respectively^37, 38^. For both infections, these strains showed a differential pathogen-sensitivity, which appeared mainly associated with variations in the AMP-mediated immune response. The resistance to *E*. *mundtii* infection seemed to be related to the production of specific AMP types, while the resistance to *S*. *marcescens* was more likely linked to a general capability to activate AMP transcription at a systemic level.

## Results and Discussion

### The four geographical strains show different survival profiles to *E*. *mundtii* and *S*. *marcescens* oral infections

**Figure 1:**
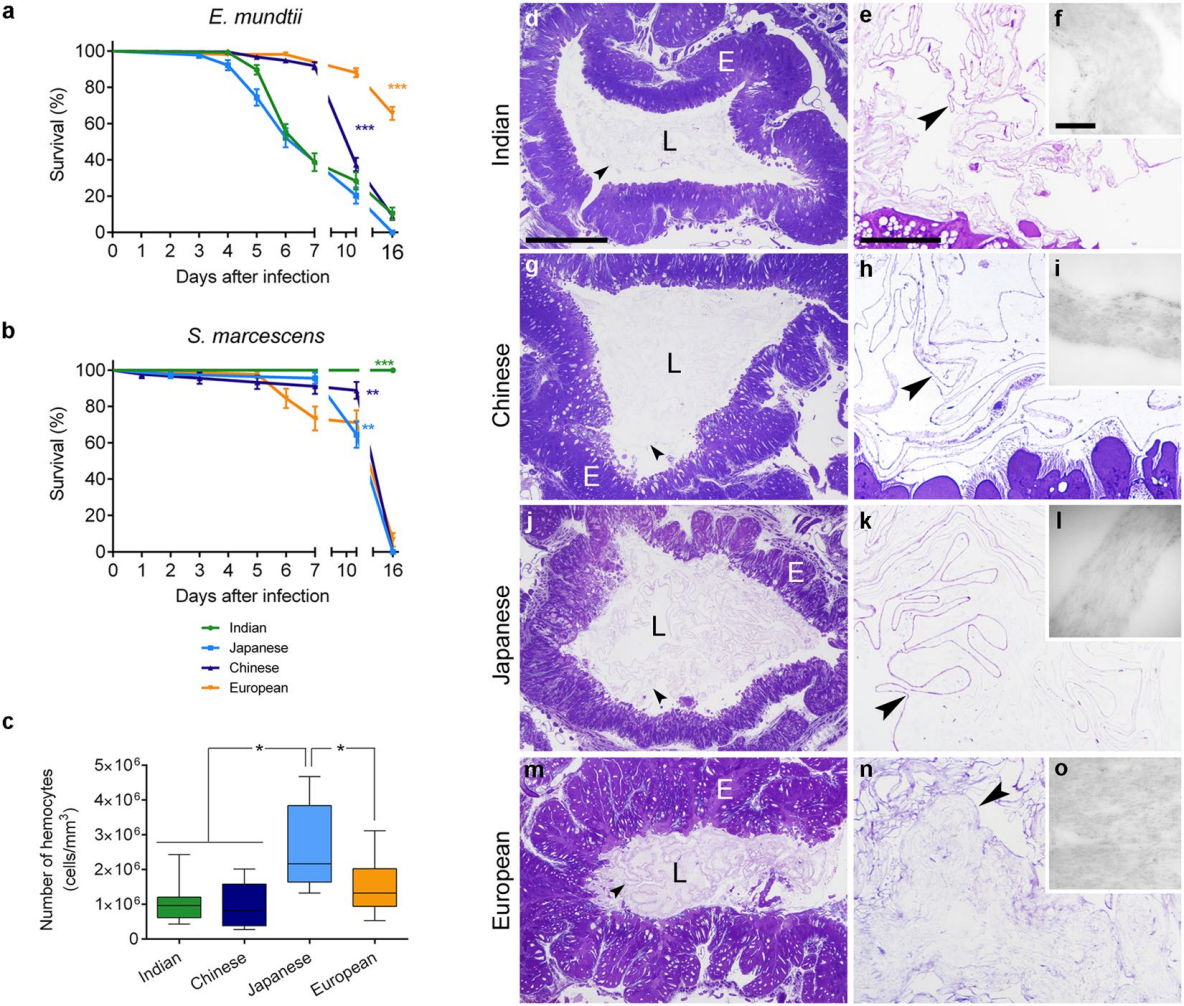
Differential sensitivity to *E*. *mundtii* and *S*. *marcescens* of the four *B*. *mori* strains. Survival curves (mean %s ± SEM) of the four *B*. *mori* strains, infected with *E*. *mundtii* (**a**) or *S*. *marcescens* (**b**). (**a**) *E*. *mundtii* strain sensitivity in Mantel-Cox test: Japanese = Indian (p = 0.11, not significant, ns) > Chinese (p < 0.0001) > European (p < 0.0001). (**b**) *S*. *marcescens* strain sensitivity in Mantel-Cox test: Japanese *vs* European (p = 0.51, ns); Japanese *vs* Chinese (p = 0.013); European *vs* Chinese (p = 0.26, ns); Indian *vs* all the other strains (p < 0.0001). ***p < 0.0001, **p = 0.013. (**c**) Number of hemocytes (Median and 10–90 percentiles of ten samples per strain) in the four *B*. *mori* strains, at the moment of the oral infection. One-way ANOVA revealed significant differences in the Japanese stain compared to all the other strains (F_3,36_ = 8.86, p = 0.0002; *Newman-Keuls *post*-*hoc* test p < 0.05). (**d**–**o**): Morphology of intestinal *epithelia* and PMs of the Indian (**d**–**f**), Chinese (**g**–**i**), Japanese (**j**–**l**) and European (**m**–**o**) strains. Light (**d**,**e**,**g**,**h**,**j**,**k**,**m**,**n**) and transmission electron (**f**,**i**,**l**,**o**) microscopy images of midgut cross sections. (**e**,**h**,**k**,**n**) are details at higher magnification of (**d**,**g**,**j**,**m**), respectively. Arrows: PM; E: midgut epithelium; L: midgut lumen. Bar in (**d**) represents 200 μm for (**g**,**j**), and (**m**); in (**e**) represents 10 μm for (**h**,**k**), and (**n**); in (**f**) represents 500 nm for (**i**,**l**,**o**).

The *E*. *mundtii* or *S*. *marcescens* sensitivities were evaluated in four *B*. *mori* strains originating from India, China, Japan and Europe and reared in germ-free conditions, which allowed the study of each single bacterium in gastrointestinal tracts not colonised by other microorganisms. The *SGIII*, *SCI*, and *Romagna bis* strains (henceforth indicated Japanese, Chinese, and European, respectively) were monovoltine (one reproductive cycle per year), the *Nistari* one (henceforth indicated Indian) was polyvoltine (i.e. multiple cycles per year). For each strain, larvae at the beginning of the fifth instar were subject to a 24 h oral exposure to each pathogen. Silkworms were then transferred to a germ-free diet and monitored until adult eclosion (Fig. 1a,b). Comparable numbers of uninfected larvae were reared in parallel as a control (Supplementary Fig. S1).

The *E*. *mundtii* infection had a significant effect on the survival rates of all four *B*. *mori* strains (Fig. 1a). However, a variable sensitivity among these geographical groups was observed. The European strain was the most resistant, with mortality occurring from the end of the fifth larval instar (days six and seven) and ~70% of individuals reaching the adult stage. The Chinese strain showed a survival profile similar to that of the European one during the larval stage. Nonetheless, several silkworms died during pupation and only 10% of individuals survived as adults. The Indian and Japanese strains were the most susceptible, showing mortality from the fourth day after exposure and only 0–10% of larvae were able to develop into adults. After *S*. *marcescens* infection, larvae belonging to the European, Chinese and Japanese strains were not able to reach the adult stage (Fig. 1b). However, Indian silkworms successfully counteracted the *S*. *marcescens* infection, since their survival rate was not significantly affected by pathogen exposure (Fig. 1b and Supplementary Fig. S1). These data indicated that the four silkworm strains were characterised by a differential sensitivity to infections, which was both pathogen- and host-dependent.

In Insects, a crucial aspect in the development of a septic infection after oral exposure is the transit of bacterial cells from the gut to the hemolymph. We therefore evaluated the presence of living bacteria as Colony Forming Units (CFUs) in hemolymph samples of infected individuals during the fifth larval instar (Table 1). *E*. *mundtii* cells were isolated in all *B*. *mori* strains. However, in the Japanese larvae, bacterial cells were found from the second day after exposure. In the Indian and European individuals, comparable CFU numbers were detected at least one day later. The bacterial load in the Chinese larvae was lower than those of the other strains (Table 1). Recently, the distinction between resistance and tolerance has been introduced also in the study of invertebrate immunity. In particular, resistance refers to the capacity to clear pathogens, while tolerance is the ability to survive despite the presence of microbes^39^. Under this perspective, the Chinese strain appeared resistant to *E*. *mundtii* in the first phases after microbial exposure, to die later during the infection. Conversely, the European strain seemed characterised by tolerance mechanisms, allowing its survival despite a high hemolymph bacterial titer.

**Table 1:** Living bacteria in the hemolymph of the four *B*. *mori* strains after oral infection.

Day after exposure	Uninfected controls				*E*. *mundtii* infection				*S*. *marcescens* infection			
	I	C	J	E	I	C	J	E	I	C	J	E
0	0	0	0	0	0	0	0	0	0	0	0	0
1	0	0	0	0	0	0	2.5 ± 2.5	1.0 ± 0.7	0	0	0	0
2	0	0	0	0	0	1.0 ± 1.0	223 ± 87	0	0	0	0	0
3	0	0	0	0	1143 ± 452	3.2 ± 2.0	3120 ± 1000	2670 ± 935	0	0	357 ± 250	0
4	0	0	0	0	1578 ± 776	0	3200 ± 1110	3210 ± 1070	0	1084 ± 1067	1428 ± 1010	385 ± 348
5	0	0	0	0	1318 ± 586	8.6 ± 5.2	>4 * 10^3^	3287 ± 998	0	>4 * 10^3^	nd	646 ± 500
6	0	0	0	0	2395 ± 526	56 ± 46	>4 * 10^3^	3234 ± 1052	0	nd	>4 * 10^3^	720 ± 408

CFUs detected in 100 µl hemolymph are shown as Mean ± SEM or >4 * 10^3^; nd: not determined; I: Indian, C: Chinese, J: Japanese, E: European.

Viable *S*. *marcescens* cells were isolated from the third day after oral exposure in the Japanese strain and from the fourth day in the Chinese and European ones (Table 1). The Indian strain did not show living bacteria in any of the collected samples, suggesting it was resistant to *S*. *marcescens*.

### Morphology of the gastrointestinal barrier and numbers of hemocytes in the four silkworm strains just before infection

The first defence line during oral infections is the gastrointestinal barrier, i.e. the peritrophic matrix (PM) and the intestinal epithelium. To evaluate whether the variable survival rate of the four silkworm strains was associated with differences in these structures, midgut samples were collected from larvae just before infection and morphologically characterised (Fig. 1d–o). The intestinal epithelium displayed comparable morphology and thickness in all the strains (Fig. 1d,g,j,m) while the PM showed some differences. The European larvae possessed a thick PM, which had a felt-like appearance and chitin fibrils aligned in dense bundles (Fig. 1n,o). The Indian, Chinese and Japanese strains showed thin PMs formed by narrow and separate layers of chitin fibrils (Fig. 1e,f,h,i,k,l).

Although PM is an essential component of the insect gastrointestinal tract, its role during oral infections is still controversial. A damaged PM conferred a higher resistance to oral exposure in *tsetse* flies, suggesting that a thick and intact PM represents a niche for bacterial proliferation^40^. Conversely, *Drosophila* with an altered PM was more susceptible to bacterial infections, indicating the importance of an intact PM in the defence against pathogens^14^. Our data indicated that in *B*. *mori* the PM thickness did not exert a fundamental role in the protection against *E*. *mundtii* or *S*. *marcescens*, since both bacteria were able to cross the PM and intestinal epithelium of the European individuals with the same temporal dynamics of other silkworm strains (the Indian for *E*. *mundtii* infection and the Chinese for *S*. *marcescens* exposure; Table 1).

To evaluate whether the variable pathogen-sensitivity was related to differences in the cellular component of the immune system, we determined the number of hemocytes in the four strains just before microbial exposure (Fig. 1c). Comparable hemocyte levels were found in the Chinese, Indian and European larvae, while the highest number was detected in the Japanese strain, which was the most sensitive to both pathogens. The hemocyte count did not therefore correlate with the differential survival profiles to both infections. In *Drosophila*, several uninfected strains were characterised by variable levels of hemocytes, which increased proportionally with the progression of septic infections with *E*. *coli* or *Enterococcus faecalis* ^16^. However, both initial cell numbers and increments during infections were not strictly linked to an enhanced pathogen-resistance^16^. It seemed therefore unlikely that variations in hemocyte numbers during infections could play a fundamental role in the differential silkworm sensitivity to *E*. *mundtii* or *S*. *marcescens*. Thus, we focused on several humoral immune components and their activation during the initial phases of the infections.

### Local and systemic AMP transcriptional activation in the four *B*. *mori* strains during oral infections with *E*. *mundtii* and *S*. *marcescens*

AMPs are the most important players of the insect humoral immunity, promoting a long-lasting defence response^41^. Quantitative real-time PCR (qPCR) experiments were performed in order to investigate whether the variable silkworm sensitivity to Gram-positive and -negative bacteria was associated with a strain-specific differential expression of AMPs at local (midgut) and/or systemic (fat body) levels. Midgut and fat body tissues were analysed for three and six days after pathogen exposure, respectively. To obtain an overview of the capacity of the infected strains to activate AMP expression, we evaluated ten among the most studied *B*. *mori* AMPs: *att*, *cec A*-*E*, *def A*-*B*, *glv 2*, *leb*, and *mor* ^22, 26^. In uninfected controls, all AMPs displayed low transcriptional levels during development (Supplementary Fig. S2). Slight expression increments were detected for some genes during the late phases of the fifth larval stage (Supplementary Fig. S2; Table S1). The basal AMP transcriptional levels of the four strains, just before infection, were compared (Supplementary Fig. S2). Significant inter-strain differences were found in the midgut for *att*, *cecB*, *cecE*, *defA glv2*, and *leb*, with the European strain often showing the highest levels. This transcriptional activity however was not sufficient to delay systemic infections on the part of either pathogen in European larvae in comparison to the other strains (Table 1). In the fat body, sporadic inter-strain variations were detected only for few genes and none of the four silkworm strains was characterised by relevant differences in the general AMP basal mRNA level at the moment of pathogen exposure (Supplementary Fig. S2).

**Figure 2:**
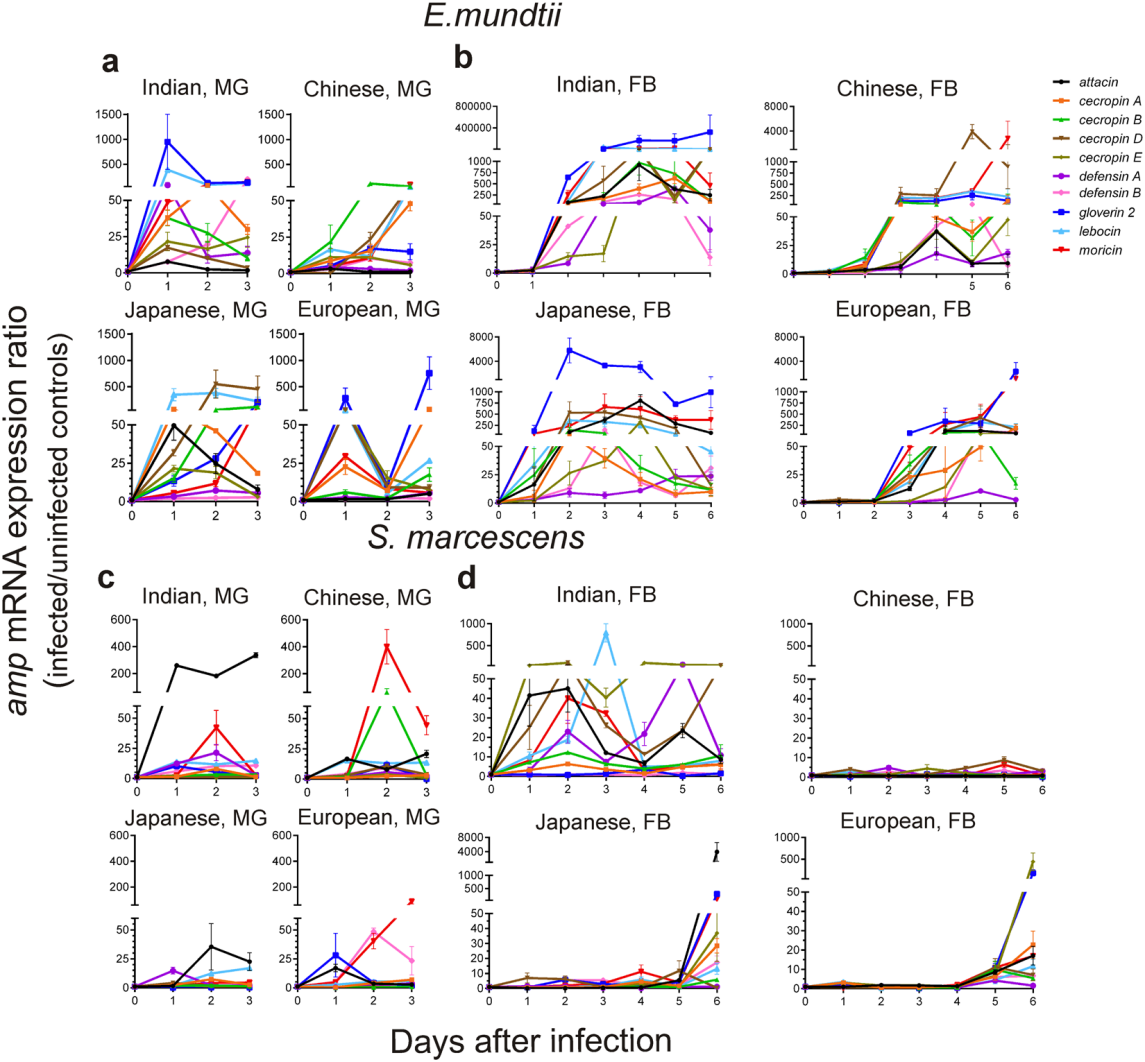
Local and systemic AMP expression induction in the four *B*. *mori* strains during *E*. *mundtii* or *S*. *marcescens* infections. AMP mRNA expression (mean ± SEM, nine samples pooled in three replicates per time per condition) was monitored for three (Midgut, MG) and six days (Fat bodies, FB) after a 24 h oral infection with *E*. *mundtii* (**a**,**b**) or *S*. *marcescens* (**c**,**d**). AMP expression ratio represents the AMP/*actin3* mRNA levels of infected samples over those of their relative uninfected controls. All AMP genes showed significant variations in expression ratio during time (p < 0.05 in Kruskal-Wallis test), except for *def B* in the European strain in (**a**), *cec B* in the Chinese strain in (**b**), *att*, *leb* and *mor* in the Chinese strain in (**d**) (statistical details in Supplementary Table S1).

During *E*. *mundtii* infection, the four silkworm populations activated the midgut AMP expression, although with some differences in the transcriptional profile kinetics (Fig. 2a; Supplementary Table S1). These data indicate that all the strains were able to sense the pathogen at a local level. In the fat body, the *E*. *mundtii* infection induced AMP expression in all the strains (Fig. 2b; Supplementary Table S1). The Indian larvae displayed the highest transcriptional activation, with increments in the expression ratio of ~five orders of magnitude. The other three strains exhibited expression increments of two-three orders of magnitude (Fig. 2b). There was a general inverse correlation between the timing of the systemic AMP induction and the susceptibility to *E*. *mundtii*. The sensitive Japanese and Indian strains activated AMP transcription in the fat body within 24–48 h after bacterial challenge, while the *E*. *mundtii*-tolerant European strain induced the AMP expression 24–48 h later (Fig. 2b).

For each strain, we determined the daily relative contribution of each AMP transcript both just before bacterial exposure and during *E*. *mundtii* infection (Supplementary Fig. S3). In all strains, the two most represented mRNAs were *cec A* and *leb* in the midgut, and *leb* and *cec B* in the fat body, at all time-points. In addition, in the midgut of European larvae, *def A* was always well-represented, and elevated relative amounts of *att* were detected at the third day of infection. At the fat body level, high percentages of *def B* seemed to be characteristic of Indian individuals, while nothing peculiar was evident in the AMP mRNA distribution of the other three strains (Supplementary Fig. S3).

Following infection with *S*. *marcescens*, the four silkworm strains showed local AMP transcription induction, albeit less pronounced than following infection with *E*. *mundtii*, suggesting they were all able to sense the pathogen (Fig. 2c; Supplementary Table S1). At the fat body level, the three *S*. *marcescens*-sensitive strains showed an enhanced expression mainly during the late stages of larval infection (Fig. 2d; Supplementary Table S1). Conversely, the resistant Indian strain was able to induce an immediate and massive AMP response from the first day after exposure (Fig. 2d; Supplementary Table S1).

As for *E*. *mundtii*, in all strains the most represented AMP mRNAs were *cec A* and *leb* in the midgut and *leb* and *cec B* in the fat body, both just before infection and in *S*. *marcescens*-infected samples (Supplementary Fig. S3). The Indian strain showed high relative amounts of *att* and *def B* transcripts at local and systemic levels, respectively. Finally, *cecD* was highly represented in the fat body of Japanese individuals, during the first three days after exposure.

### The hemolymph antimicrobial activity parallels the differential silkworm sensitivity to both *E*. *mundtii* and *S*. *marcescens* infections

**Figure 3:**
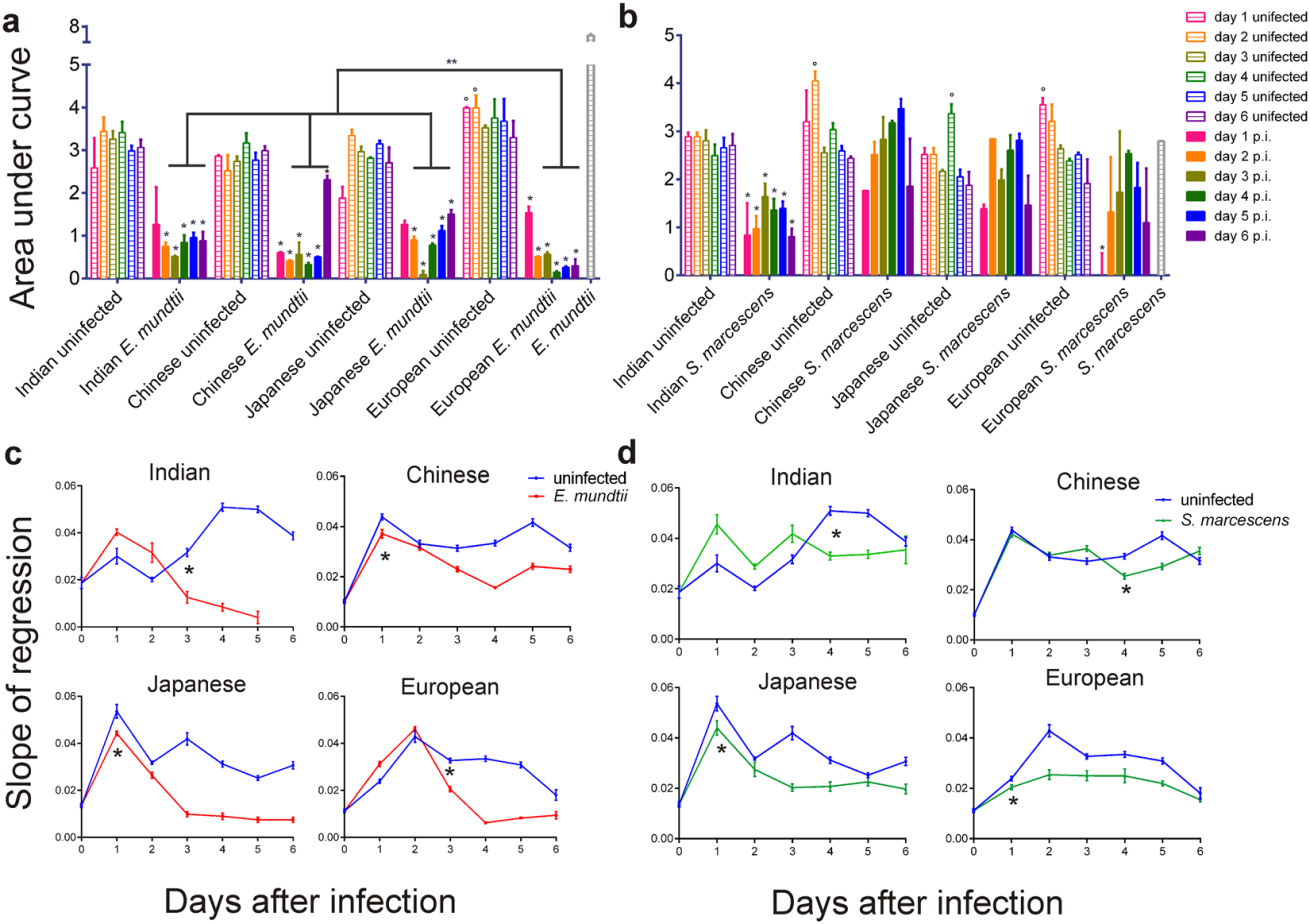
Plasma antimicrobial activity and melanization rate in the four *B*. *mori* strains during six days after *E*. *mundtii* or *S*. *marcescens* infections. (**a**,**b**) *In vitro E*. *mundtii* (**a**) or *S*. *marcescens* (**b**) growth in *E*. *mundtii* (**a**) or *S*. *marcescens* (**b**) infected plasma and relative uninfected controls, for six days after bacterial challenge. The bacterial growth is evaluated as Area under curve between 3 and 9 h *post*-*inoculum* (mean ± SEM; nine plasma samples in three replicates per time per condition); p.i.: post-infection. In (**a**) two-way ANOVA revealed significant variations in infected *vs* uninfected samples for each strain (effect of condition: p < 0.0001 for all strains). *Indicates significant differences between infected *vs* uninfected samples from the same day within the same strain (Bonferroni *post*-*hoc* test; p < 0.05). **Indicates significant differences between infected plasma of the different strains (two-way ANOVA p < 0.0001). In (**b**) two-way ANOVA revealed significant variations in infected *vs* uninfected samples in the Indian strain (effect of condition: p < 0.0001) and in the plasma from the first day after infection in the European strain (p < 0.005). *Indicates significant differences between infected *vs* uninfected samples from the same day (Bonferroni *post*-*hoc* test; p < 0.05). In (**a**,**b**) °indicates sporadic significant differences among samples from the same day in uninfected strains (statistical details in Supplementary Tables S2 and S3); (**c**,**d**) Melanization rate of *E*. *mundtii* (**c**) or *S*. *marcescens* (**d**) infected plasma and relative uninfected controls, for six days after bacterial challenge. Slopes of melanization regression lines are calculated for each condition (mean ± SEM; nine plasma samples in three replicates per time per condition). Two-way ANOVA revealed significant variations in infected *vs* uninfected samples for each strain [effect of condition: p < 0.0001 for all the four strains (statistical details in Supplementary Table S4 and S5)]. *Significant lower melanization rates in infected plasma compared to the relative uninfected controls evaluated in Bonferroni *post*-*hoc* test (p < 0.05).

AMPs produced by the fat body are known to be released in the hemolymph^1, 5^. We explored whether the variations in AMP expression observed in infected strains reflected a different antimicrobial activity in the hemolymph. Plasma samples from infected and uninfected silkworms were added to *E*. *mundtii* or *S*. *marcescens* cultures and bacterial growth (μ) was followed for the subsequent 24 h (Supplementary Fig. S4). To compare the growth kinetics in the different conditions, we calculated the area under the μ curve for each sample. Uninfected plasma was found to provide a good substrate for microbial proliferation for both bacteria, with some sporadic variations related to the silkworm strain or the day of collection (Fig. 3a,b; Supplementary Tables S2 and S3). Most of the *E*. *mundtii*-infected samples contained active antimicrobials since they induced a significant reduction of *E*. *mundtii* growth *in vitro* (Fig. 3a; Supplementary Table S2). In all the strains, the plasma extracted from larvae at the first day after *E*. *mundtii* exposure exhibited a less pronounced antimicrobial activity, when compared to that from the other time-points. This might be due to a slow kinetics in the production of active AMPs at the fat body level in the initial phase of the immune response, in which case the observed antimicrobial activity could be related to the contribution of other tissues, such as hemocytes, known to be involved, to a lesser extent, in AMP synthesis^42^. Excluding the first day, the overall plasma antimicrobial activity of the European strain was significantly higher compared to those of all the other strains, which showed similar antimicrobial levels (Fig. 3a; Supplementary Table S2). This was particularly evident for samples derived from larvae at the sixth day after exposure.

The plasma derived from *S*. *marcescens*-infected silkworms belonging to the Chinese and Japanese strains did not show any significant antimicrobial activity when tested against *S*. *marcescens* (Fig. 3b; Supplementary Table S3). The European population showed a significantly higher antimicrobial activity only in samples derived from the first day after microbial challenge, while infected plasma from all the other time-points showed an antimicrobial activity similar to those of their relative uninfected controls (Fig. 3b; Supplementary Table S3). These data were consistent with the limited transcriptional induction of AMPs detected at the fat body level in the three strains. In the Indian population, which showed high systemic AMP transcription, significant antimicrobial activity was observed in all infected plasma samples when compared to uninfected controls (Fig. 3b; Supplementary Table S3).

### Melanization contributes to the differential silkworm response against *S*. *marcescens*

During infections, melanin is rapidly produced and accumulates on foreign microbes, contrasting their growth and mediating phagocytic cell activities^43^. Some pathogens are able to interfere with melanin synthesis^28^. For each silkworm strain, we measured the melanization rate of plasma samples, collected every 24 h after the challenge with *E*. *mundtii* or *S*. *marcescens* (Fig. 3c,d; Supplementary Figs S5 and S6). For both infections, all the strains showed significant variations in infected samples compared to uninfected controls. However, there were some differences between the two types of microbial exposures.

During *E*. *mundtii* infection, the melanization reaction was inhibited from the third day in all *B*. *mori* strains, suggesting that the pathogen interfered with melanin synthesis (Fig. 3c). Significant increments were detected during the first two days of the infection in the European (the most tolerant) and Indian (one of the most sensitive) strains (Supplementary Table S4). It seemed therefore unlikely that the melanization response was related to *E*. *mundtii* strain-specific susceptibility.

After *S*. *marcescens* exposure, melanization was inhibited from the first day of the infection in the European and Japanese individuals and from the fourth day in the Indian and Chinese larvae (Fig. 3d). These data suggest that also *S*. *marcescens* was able to interfere with melanin production. However, during the first phase after bacterial exposure, melanization rate showed significant increments exclusively in the *S*. *marcescens*-resistant Indian individuals, while the *S*. *marcescens*-sensitive strains displayed similar or lower melanization in comparison to uninfected controls (Supplementary Table S5). The melanization response might represent one of the immune components involved in *B*. *mori* defence against *S*. *marcescens*.

Since lysozyme is an additional humoral component possibly involved in differential pathogen-sensitivity, we measured lysozyme levels in plasma samples derived from *E*. *mundtii*- or *S*. *marcescens*-infected larvae and uninfected controls (Supplementary Table S6). Both *E*. *mundtii*- and *S*. *marcescens* exposures modified lysozyme activities of the four *B*. *mori* strains. However, since similar variations were observed in resistant/tolerant and sensitive strains during both infections, the lysozyme contribution did not appear to be directly associated with the differential silkworm sensitivity profiles.

### *B*. *mori* AMP gene variability and protection against *E*. *mundtii* infection

With the progression of *E*. *mundtii* infection, European larvae showed the highest plasma antimicrobial activity in comparison to those of the other strains. Since all the *B*. *mori* strains were able to activate fat body AMP transcription, one of the possible factors contributing to the differential pathogen-sensitivity could be linked to the composition of the AMP cocktail (i.e. showing variability in the relative abundance of particular AMP classes and/or isoforms). Distinct AMP types are known to show different *in vitro* antimicrobial activity^22, 44^. However, the systemic transcriptional profiles of the ten representative AMPs did not show any evident strain-specific pattern which might explain the lower *E*. *mundtii* susceptibility of European individuals. Considering AMPs within the same class, the differential pathogen sensitivity could be linked to the presence of strain-specific AMP isoforms with a variable antimicrobial activity.

For each strain, we sequenced the 23 AMP coding genes, known to be expressed in *B*. *mori* after immune challenge^22–26^. Several single nucleotide polymorphisms (SNPs) were identified in 16 AMP genes, while *cec A1*, -*A2*, -*B1*, -*B2*, -*B4*, -*B5*, and *mor* were characterised by a unique gene sequence, common to all four silkworm strains (Table 2; Supplementary Table S7). Polymorphic alleles were detected in the Chinese, Japanese and European strains, while the Indian population was monomorphic in all the analysed genes. Given that domestication is generally associated with a reduction in heterozygosity^29^, these data might be due to the rearing conditions that the different strains have experienced at the CREA sericulture seat. The Indian population is mostly used as a laboratory strain and maintained through small reproductive pools, while the Japanese, Chinese and European strains are used for commercial purposes and reared in larger populations.

**Table 2:** AMP alleles and isoforms in the four *B*. *mori* strains.

Gene	cds (bp)	AMP (aa)	Active AMP (aa position)	N of alleles	S	NS	NS (act)	N of isoforms	N of mature isoforms
*att 1*	645 bp	214 aa	188 aa (27–214)	3	13	—	—	1	1
*att 2*	645 bp	214 aa	188 aa (27–214)	2	6	1	1	2	2
*cec A1*	192 bp	63 aa	35 aa (27–61)	1	—	—	—	1	1
*cec A2*	192 bp	63 aa	35 aa (27–61)	1	—	—	—	1	1
*cec B1*	192 bp	63 aa	35 aa (27–61)	1	—	—	—	1	1
*cec B2*	192 bp	63 aa	35 aa (27–61)	1	—	—	—	1	1
*cec B3*	192 bp	63 aa	35 aa (27–61)	2	—	1	1	2	2
*cec B4*	192 bp	63 aa	35 aa (27–61)	1	—	—	—	1	1
*cec B5*	192 bp	63 aa	35 aa (27–61)	1	—	—	—	1	1
*cec B6*	192 bp	63 aa	35 aa (27–61)	3	1	1	1	2	2
*cec D*	186 bp	61 aa	37 aa (25–61)	2	1	—	—	1	1
*cec D2*	201 bp	66 aa	?	3	1	2	1 (?)	3	2 (?)
*cec E*	198 bp	65 aa	?	2	5	—	—	1	1
*def A*	279 bp	92 aa	36 aa (57–92)	2	—	1	—	2	1
*def B*	249 bp	81 aa	38 aa (44–81)	4	4	6	1	4	2
*enb 1*	180 bp	59 aa	37 aa (22–58)	2	6	4	4	2	2
*enb 2*	180 bp	59 aa	37 aa (22–58)	3	8	4	4	2	2
*glv 1*	537 bp	178 aa	135 aa (44–178)	5	12	5	2	4	3
*glv 2*	522 bp	173 aa	131 aa (43–173)	4	13	4	1	3	2
*glv 3*	519 bp	172 aa	131 aa (42–172)	4	13	6	1	4	2
*glv 4*	516 bp	171 aa	131 aa (41–171)	3	6	3	—	3	1
*leb*	540 bp	179 aa	32 aa (121–152)	2	2	4	—	2	1
*mor*	201 bp	66 aa	42 aa (25–66)	1	—	—	—	1	1

cds: length of the coding sequence; S: number of synonymous substitutions; NS: number of non-synonymous substitutions; NS (act): number of non-synonymous substitutions in the active portion of the peptide; (?): missing information on the precise position of the active sequence of the peptide.

About one-third of the SNPs were non-synonymous substitutions which modified the AMP aa sequences (Table 2; Supplementary Table S7). From two to four peptide isoforms were identified for 13 AMPs, while Att 1, Cec D, and Cec E showed the same aa sequence in all the strains. In some cases, the aa modification mapped to the signal peptide or within the portion which is removed during AMP maturation (Table 2; Supplementary Table S7). It is unlikely that these modifications have a direct effect on AMP activity, although they might be important in the regulation of the AMP maturation process.

Non-synonymous substitutions were identified in the active portion of Att 2, Cec B3, -B6, Def B, Enb 1, -2, Glv 1, -2, and -3, and originated two isoforms for each mature AMP, with the exception of Glv 1, that showed three variants (Table 2; Supplementary Table S7). Due to the high level of conservation among *cec B1*-*6* paralogues, Cec B3 and Cec B6 isoforms 1 (iso 1) had the same aa sequence, although encoded by different alleles mapping to two distinct genes (Table 2; Supplementary Table S7). High similarity levels also characterised the two *enb* paralogues, therefore Enb 1 iso 1 and −iso 2 were identical to Enb 2 iso 1 and −iso 2, respectively.

The different isoforms were compared *in silico* using AMPA^45^, CAMP^46^ and APD3^47^ prediction tools, which are able to estimate the antimicrobial potential of a given peptide (Table 3). For Att 2, Cec B3, Def B, Glv 2, and -3, the detected isoforms showed small variations in the output values, suggesting they possessed similar antimicrobial activity. In addition, the frequency distributions of their isoforms did not appear to be related to the differential *E*. *mundtii* sensitivity (Table 3). They were therefore excluded from subsequent analyses. Enb 1/2 iso 1 was predicted to be more hydrophobic compared to iso 2, while the latter resulted more thermostable and with a higher antimicrobial potential (Table 3). However, the two Enb1/2 isoforms were both detected in the Indian (iso 1, 100%) and Japanese (iso 2, 100%) strains, which showed a similar *E*. *mundtii* sensitivity (Fig. 1a). It seemed therefore unlikely that a different antimicrobial activity of the Enb1/2 variants could represent a key factor in the differential sensitivity to *E*. *mundtii*.

**Table 3:** Antimicrobial activity prediction of the mature AMP isoforms detected in the four *B*. *mori* strains.

AMP	N	Frequency (%)				aa substitutions	AMPA	CAMP/APD3				
		I	C	J	E		P	Charge	B Index	A Index	Hydropathy	P
Att 2	1	100				81 V		+3	1.38	70.16	−0.26	
	2		100	100	100	81I		+3	1.38	70.69	−0.26	
Cec B3	1		100	100	100	G38, E53		+7	1.45	100.27	−0.24	
	2	100				S38, E53		+7	1.57	100.27	−0.25	
Cec B6	1	100	100	100		G38, E53		+7	1.45	100.27	−0.24	
	2				100	G38, Q53	+	+8	1.42	100.27	−0.24	+
Def B	1	100				Y61		+3	1.95	43.68	−0.62	
	2		100	100	100	H61		+3	2.07	43.68	−0.67	
Enb 1/2	1	100	83		58	F25 G51 A54 A55		+1	0.79	87.63	0.41	
	2		17	100	42	I25 A51 T54 S55	+	+1	0.97	95.26	0.38	+
Glv 1	1	100	58	100	42	K53 G57		−4	1.84	62.89	−0.61	
	2		35			R53 G57		−4	1.91	62.89	−0.62	
	3		6		58	K53 R57	+	−3	1.96	62.89	−0.64	+
Glv 2	1	100			40	K74		0	2.11	55.11	−0.80	
	2		100	100	60	R74		0	2.18	55.11	−0.80	
Glv 3	1	100	70	70	45	R49		−1	2.24	58.85	−0.75	
	2		30	30	55	K49		−1	2.17	58.85	−0.75	

Frequency (%): frequencies of the isoforms in the four strains (I: Indian, C: Chinese, J: Japanese, E: European); AMPA: output of the AMPA tool; CAMP/APD3: output of the CAMP/APD3 tools; Charge: net charge; B Index: Boman index; A Index: Aliphatic Index; P: antimicrobial prediction; +: indicates for each AMP the isoform with the highest predicted antimicrobial activity.

The Cec B6 iso 2, characterised by a Q in position 53, displayed a higher propensity to be antimicrobial compared to the iso 1, carrying an E in the same position. Moreover, Q53 Cec B6 iso 2 had a more positive net charge (+8 *vs* +7), which is an important factor in the interaction between cationic AMPs and negatively charged bacterial membranes. Similarly, Glv 1 iso 3 had the highest predisposition to be antimicrobial and the least negative net charge (−3 *vs* −4) in comparison to the other two isoforms, which showed similar predicted antimicrobial activities (Table 3).

### The Q53 Cecropin B6 isoform contributes to the protection against *E*. *mundtii* infection

**Figure 4:**
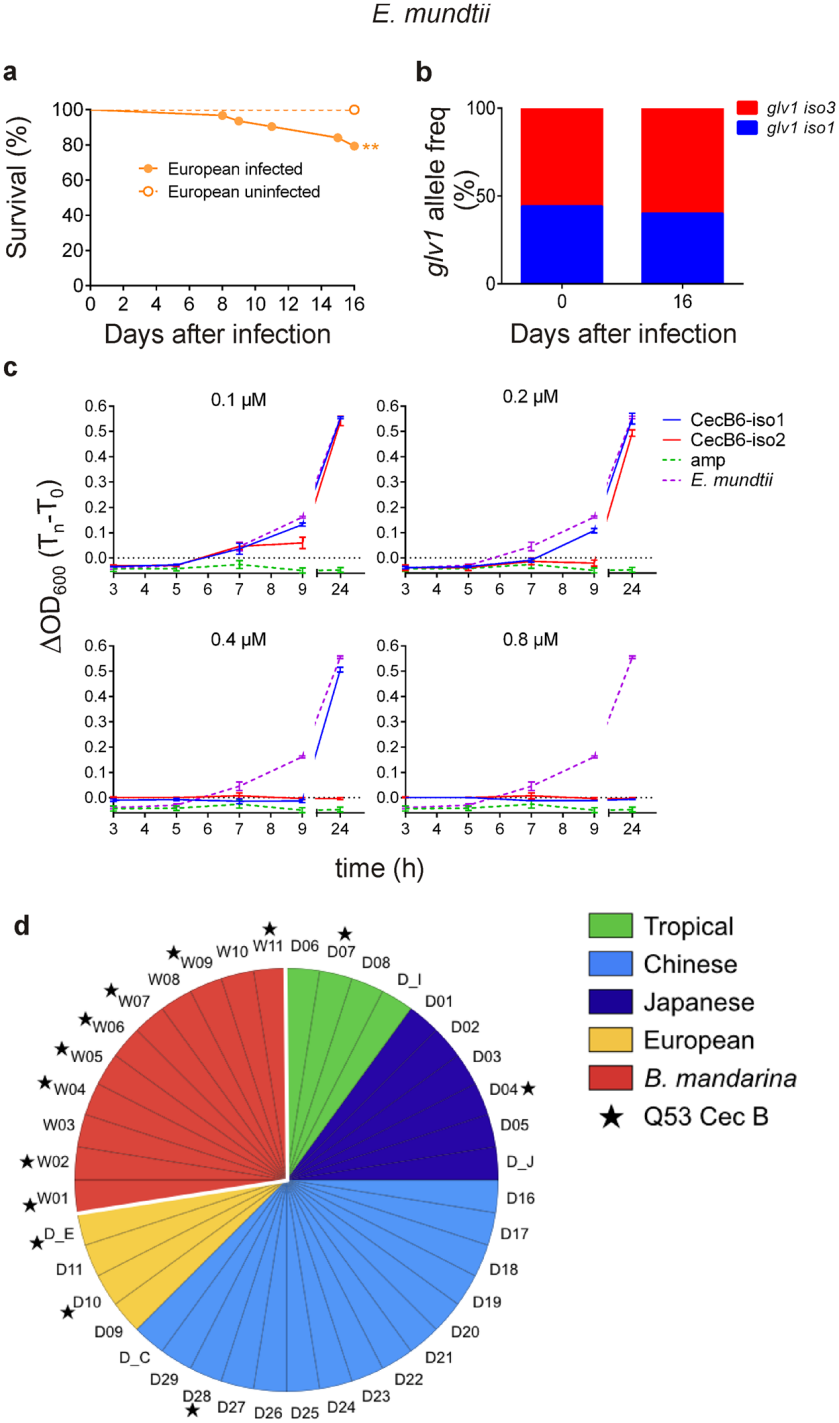
Contribution of *B*. *mori* Glv 1 and Cec B6 isoforms to the protection against *E*. *mundtii*. (**a**) Survival curves of sixty-three European larvae orally infected with *E*. *mundtii* and seventy uninfected controls (**p = 0.0013 in Mantel-Cox test). (**b**) *glv 1* ^*iso1*^ (blue) and *glv 1* ^*iso3*^ (red) allelic frequencies before *E*. *mundtii* infection (day 0) and in adult survivors (day 16). (**c**) MIC of E53 Cec B6 iso1 (blue) and Q53 Cec B6 iso2 (red) evaluated at 0.1, 0.2, 0.4 and 0.8 µM against *E*. *mundtii*. ΔOD600 (T_n_-T_0_; mean ± SEM): differences in OD_600_s obtained at time 0 (T_0_) with those measured at different time points (T_n_: 3, 5, 7, 9, and 24 h). amp (green): negative control, bacterial growth in presence of ampicillin (50 µg/µL). *E*. *mundtii* (purple): positive control, bacterial growth in culture medium. (**d**) Presence of the Q53 Cec B variant in *B*. *mori* and *B*. *mandarina*. W (red area): *B*. *mandarina* silkworms; D: *B*. *mori* domesticated silkworms. The strains of this work are indicated as: D_I (Indian), D_J (Japanese), D_C (Chinese), D_E (European); all the others derived from ref. 29. Tropical region: green; Japan: purple; China: light blue; Europe: yellow.

For both Glv 1 and Cec B6, the variants with the highest putative antimicrobial potential were mostly or exclusively represented in the European strain, with a frequency of 58% for Glv 1 iso 3 and 100% for Q53 Cec B6 iso 2 (evaluated on 73 and 15 individuals, respectively). To obtain an indication about the possible involvement of Glv 1 isoforms in the protection of *B*. *mori* against *E*. *mundtii*, we evaluated the frequency variations in the alleles coding the Glv 1 iso 1 and iso 3 in a European population, during *E*. *mundtii* infection. Sixty-three larvae were exposed to the pathogen at the beginning of the fifth instar, collected immediately after death and subsequently genotyped (Fig. 4a,b). In this population, the *glv 1* ^*iso1*^ and *glv 1* ^*iso3*^ allele frequencies were respectively 44 and 56% at the beginning of the infection (i.e.: 25% *glv 1* ^*iso1*/*iso1*^, 36% *glv 1* ^*iso3*/*iso3*^ homozygous silkworms, and 38% *glv 1* ^*iso1*/*iso3*^ heterozygous individuals). In adult survivors (day sixteen) we detected a slight increase of the *glv 1* ^*iso3*^ allele which reached a frequency of 60% with a parallel decrease of the *glv 1* ^*iso1*^ allele to 40% (i.e.: 24% *glv 1* ^*iso1*/*iso1*^, 34% *glv 1* ^*iso3*/*iso3*^ homozygotes, and 41% *glv 1* ^*iso1*/*iso3*^ heterozygotes). Nonetheless, this change was not significant when evaluated as number of dead/alive individuals carrying the *glv 1* ^*iso3*^ allele in a 2X2 Contingency Table (Fisher Exact Test p = 0.7, ns). Similar results were obtained with a logistic regression analysis, in which the probability to be alive was correlated with the three genotypes (p = 0.45, ns). These data suggest a minor influence of Glv 1 iso 3 to the differential sensitivity of silkworms to *E*. *mundtii* infection.

The contribution of Cec B6 isoforms was evaluated *in vitro* by determining the Minimal Inhibitory Concentration (MIC) of the chemically synthesised Cec B6 variants against *E*. *mundtii* (Fig. 4c). The European Q53 Cec B6 iso 2 showed a 2-fold higher antimicrobial activity in comparison to E53 Cec B6 iso 1 (0.4 *vs* 0.8 μM), characteristic of the other three strains. At 0.2 and 0.1 μM, both peptides did not prevent bacterial proliferation, although Q53 Cec B6 iso 2 inhibited pathogen growth during the first 9 h more efficiently when compared to E53 iso 1 (Fig. 4c). It is important to underline that the difference observed *in vitro* might be amplified *in vivo*, since the peptide concentration sufficient to inhibit the *in vitro* bacterial growth is considerably higher than that required in the living organism^48^. These data suggest that the Q53 Cec B6 iso 2 contributed to the *E*. *mundtii*-tolerance of the European strain analysed in this study.

To have an indication on the distribution of the E53Q substitution at the level of Cec B peptides in *Bombyx*, we searched for the presence of a specific SNP (G157C) in the *cec B* nucleotide sequences of thirty-six non-assembled silkworm genomes, recently resequenced by ref. 29. Among these, twenty-five derived from *B*. *mori* geographical strains and eleven from wild silkworms belonging to the *Bombyx mandarina* species, which is considered to be the wild ancestor of *B*. *mori* (Supplementary Table S8). In both *B*. *mandarina* and *B*. *mori* genomes, most of the *cec B* sequences were characterised by a G in position 157, which gives rise to the E53 CecB isoform in the deduced aa sequences. In eight *B*. *mandarina* and four *B*. *mori* genomes, the C157 substitution, originating the Q53 Cec B variant, was identified in some *cec B* sequences corresponding to at least one *cecB* paralogue (Fig. 4d). Since Xia and colleagues sequenced one single individual per strain^29^, the 157 C polymorphism could be more widespread. Given the high similarity level among paralogues, it was difficult to determine the specific *cec B* gene carrying this modification. However, these analyses suggested that the G157C SNP was present before domestication and it has been maintained in some *B*. *mori* geographical strains.

The presence of a specific Cec B6 isoform could be only one of the possible reasons which explains the differential *E*. *mundtii*-sensitivity of the European strain analysed in our study. This association will be further explored with transgenic tools available for *B*. *mori* and in other geographical strains, carrying the same AMP variant in a different genetic background. However, it is interesting to mention that a specific amino acid polymorphism in *Drosophila* Diptericin was recently found to be highly predictive of resistance to a particular bacterial infection^15^.

### Differential activation of the immune response and protection against *S*. *marcescens* infection

The Indian population was resistant to *S*. *marcescens*, showed an early and massive AMP transcription at the fat body level and a corresponding plasma antimicrobial activity against the pathogen. The three *S*. *marcescens*-sensitive strains displayed a weak or delayed AMP transcriptional induction and no relevant antimicrobial activity at the hemolymph level.

**Figure 5:**
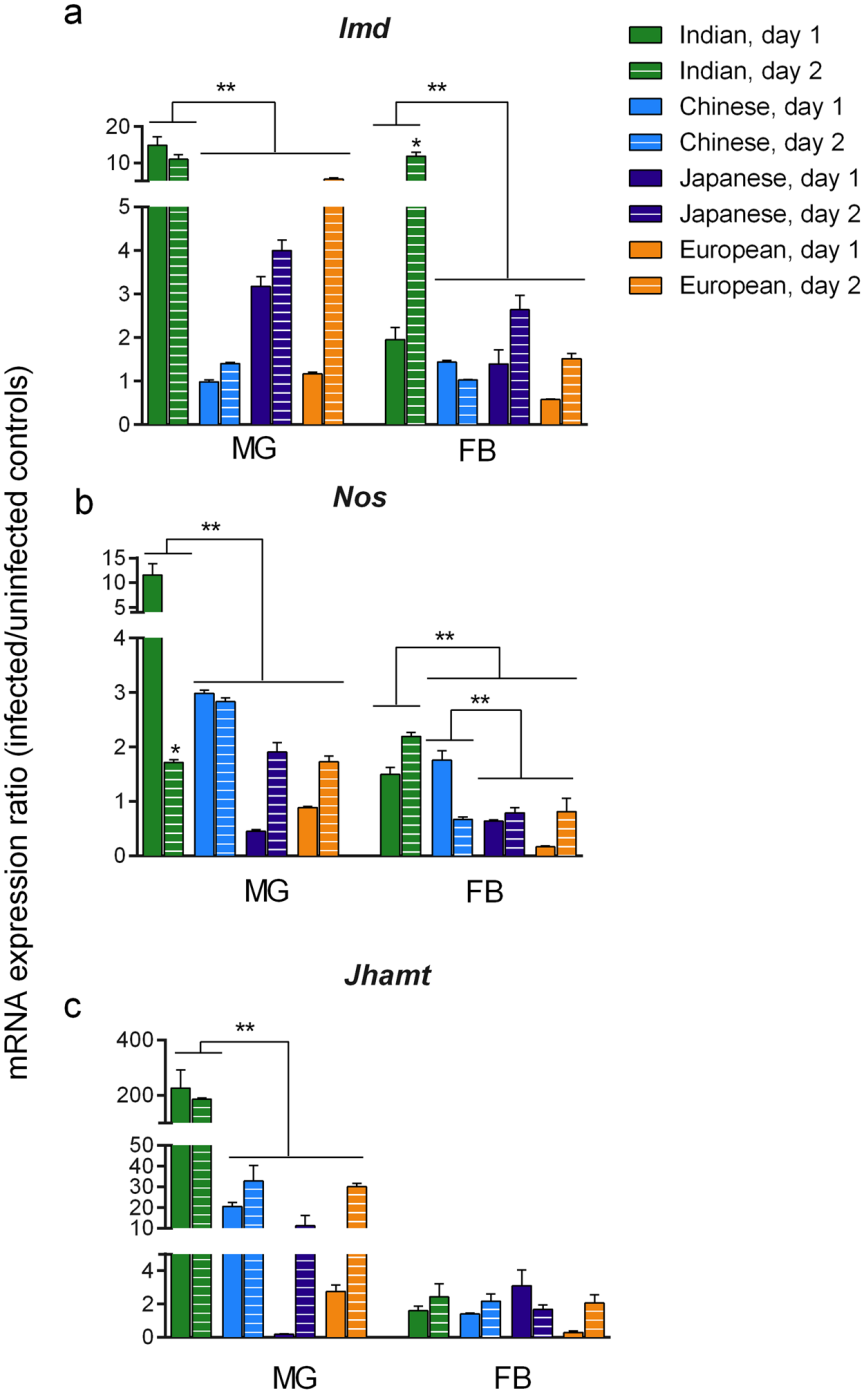
Local and systemic expression of *Imd*, *Nos* and *Jhamt* after *S*. *marcescens* infection. The mRNA expression of *Imd* (**a**), *Nos* (**b**) and *Jhamt* (**c**) in the midgut (MG) and fat bodies (FB) of the four *B*. *mori* strains for two days after *S*. *marcescens* infection. Expression ratios represent the immune gene/*actin3* mRNA levels of infected samples over those of their relative uninfected controls (mean ± SEM, nine samples pooled in three replicates per time per condition). The Indian strain showed the highest expression values for all the three genes in the midgut (two-way ANOVA, effect of strain: p < 0.0001 for each of the three genes), and for *Imd* and *Nos* in the fat body (two-way ANOVA, effect of strain: p < 0.0001). **Indicates significant differences in expression levels among the four strains (Bonferroni *post*-*hoc* test, p < 0.01); *indicates a significant intra-strain difference in the expression levels of the target gene between the first and second day (Bonferroni *post*-*hoc* test p < 0.05). (Statistical details in Supplementary Table S9).

These data suggest the presence of strain-specific differences in the activation of the immune response. We therefore determined the local and systemic expressions of three factors known to stimulate AMP induction: *Imd*, *Nitric oxide synthase 1* (*Nos*) and *Juvenile hormone acid methyltransferase* (*Jhamt*)^9, 12, 28, 49^. Transcriptional variations in midgut and fat body were compared for two days after bacterial exposure in the four strains (Fig. 5).

*Imd*, a fundamental factor of the humoral immune response cascade during Gram-negative bacterial infections, showed the highest transcriptional induction in the midgut of the Indian silkworms from the first day after microbial exposure. Among the *S*. *marcescens*-sensitive strains, a lower or delayed *Imd* local transcription was observed in Japanese and European individuals, while no significant increase was detected in Chinese silkworms (Fig. 5a; Supplementary Table S9). The differences in *Imd* mRNA induction were more evident in the fat body, where a 10-fold increase was detected from the second day after microbial exposure in the Indian strain, while from 2- to 3- fold increments were characteristic of Japanese and European silkworms and no induction was detected in the Chinese strain.

We also identified strain-specific differences in the induction profile of *Nos*. Nos is responsible for the synthesis of nitric oxide (NO), a key factor in the early step of the AMP activation pathway during Gram-negative infections^12, 49, 50^. Moreover during oral infection, NO likely represents a fundamental signal involved in inter-tissue communication which from the gut activates AMP production in the fat body^49, 50^.

All strains were able to overexpress *Nos* in the midgut, with Indian individuals showing the highest mRNA inductions (Fig. 5b; Supplementary Table S9). In the fat body, the Indian strain activated *Nos* transcription during both days, Chinese silkworms showed only a transient increase on the first day after bacterial challenge, and no induction was detected in European and Japanese individuals (Fig. 5b; Supplementary Table S9). These data, together with *Imd* induction variations, might be linked to the differential capability to induce AMPs at a systemic level shown by the four silkworm strains.

All *B*. *mori* strains activated local and systemic expressions of *Jhamt*, although Indian individuals showed the highest local mRNA induction. No significant differences were detected among strains at the fat body level (Fig. 5c; Supplementary Table S9). *Jhamt* is involved in the production of Juvenile Hormone (JH), which in *Bombyx* controls the molting behaviour and promotes the Imd pathway during infections^27, 28, 51^. These data suggest that the JH pathway was not associated with the differential capability of silkworm strains to respond to *S*. *marcescens* infections. They also indicate that the differences in the voltinism characterising the four strains did not influence the ability to induce *Jhamt* transcription, when exposed to *S*. *marcescens* pathogen.

Functional studies on the possible differential activities of the proteins involved in the induction of the immune response are still required. In particular, it has been observed that *S*. *marcescens* is able to elude the silkworm immune response by decreasing adhesive properties of hemocytes and inhibiting phagocytosis^52, 53^. Although we did not detect any clear association between the hemocyte numbers and pathogen sensitivity in the four strains, the *S*. *marcescens* resistant phenotype of the Indian population might also be linked to the activity of regulatory elements involved in the immunosurveillance process. However, our data suggest that the Indian strain possessed a more efficient activation of AMP production and a pivotal role might be played by the NO-signaling pathway, which promotes the inter-tissue communication following oral infections. AMP effectors released in the hemolymph, together with the enhanced melanization response in the first phases after microbial exposure, appear to contribute to the *Serratia*-resistant phenotype detected in the Indian strain.

## Conclusions

Sensitivity to infection is a complex genetic trait and silkworm strains with different genetic backgrounds show a variable susceptibility to pathogen exposure^34–36^. Our study indicates that distinct *B*. *mori* strains possess different mechanisms to counteract bacterial oral infections, the effectiveness of which appears to be pathogen-dependent. Such defence mechanisms seem to be based on the production of specific AMPs, such as the Q53 Cec B6 variant identified in the European strain and active against *E*. *mundtii*, or on a prompt induction of the humoral immune response, as observed in the Indian individuals during *S*. *marcescens* infection. In the fruit fly *D*. *melanogaster*, both natural populations and laboratory strains showed a variable sensitivity to pathogen infections^15–19^. Initial studies suggested that the differential ability to counteract infections relies on the efficient production of AMP cocktails with a wide antimicrobial activity^17–19^. However, recent evidence underlined that, at least in some cases, specific isoforms in the AMP mixture might also represent a key resistance factor^15, 54^. Such data indicate that in insects two immunological strategies might have evolved in the natural environment. *B*. *mori* is a completely domesticated species that has lost the capability to survive in the wild and silkworm strains are currently preserved in dedicated germplasm banks^30^. It is therefore possible that the different *B*. *mori* strains, artificially selected for their productive traits during the domestication process, might have been also (intentionally or unintentionally) selected for their ability to counteract infections through one of the two strategies. The extension of the study to other silkworm strains as well as other pathogens will help to evaluate this hypothesis. It would be equally important to evaluate the pathogen effects in controlled conditions closer to the natural feeding regime, taking into account also the contribution of the silkworm microbiota.

Finally, we have explored the genetic and molecular mechanisms at the basis of the silkworm differential susceptibility to pathogen oral exposures. While in insects the oral infection is ecologically relevant, traditionally several studies employ the systemic injection to examine both the pathogen-sensitivity and immune response activation of the host organism. However, in *Drosophila* it has been demonstrated that the genetic basis of the host susceptibility can also vary, depending on the way of infection^55^. In addition, in *B*. *mori* it has been shown that the oral administration and systemic injection differentially affect the host transcriptional activation^56^. Several factors might play a role in the possible pathogen-sensitivity variations caused by the two routes of infections, such as the intrinsically different host-pathogen interaction established by the two challenges. These differences might activate the host defence weapons with differential temporal dynamics and modalities, even when both challenge routes trigger a systemic immune response. This implies that the pathogen injection cannot be considered *a priori* just an infective route which abolishes the gastrointestinal physical barrier. Nevertheless, in studies of this kind, wherever one of the analysed challenging strategies should test negative, the complementary trial could be performed to evaluate the role of each route in affecting the induction of the immune response and, in turn, the host pathogen-sensitivity profile.

## Materials and Methods

### *Bombyx mori* strains and rearing conditions

The monovoltine strains European *Romagna bis* (Italy, selected in 1990), Chinese *SCI* and Japanese *SGIII* (imported in 1997) were maintained in the CREA germplasm bank as large populations, originating from at least 72 independent egg-layings (~400–600 eggs each), derived from single couples. The Nistari polyvoltine Indian strain was obtained in 2009 from the Institut National de la Recherche Agronomique, Unité Nationale Séricicole, France, and transferred to CREA as few layings derived from a limited number of individuals. Its reproduction was restricted to 15–20 layings of ~250–400 eggs. Silkworms were reared at 28 ± 1 °C in 12 h:12 h light:dark regime on germ-free artificial diet^57^ and manipulated under a laminar flow hood.

### Light and transmission electron microscopy

Larvae at the first day of the fifth instar were ice-anaesthetised and cut dorsally. For each strain, at least six midgut samples, including the PM, were immediately isolated and processed as in ref. 58. Semi-thin sections stained with crystal violet and basic fuchsin were observed with a Nikon Eclipse Ni-U microscope. Images were acquired with a DS-5 M-L1 digital camera system (Nikon, Tokyo, Japan). Thin sections were stained with uranyl acetate and lead citrate and observed with a JEOL JEM-1010 electron microscope (Jeol, Tokyo, Japan). Images were acquired with an Olympus Morada digital camera (Olympus, Münster, Germany).

### Count of hemocytes

For each strain, hemolymph samples were collected from the abdominal prolegs of at least 10 anaesthetised larvae at the first day of the fifth instar and fixed in formaldehyde (0.5% final concentration). Hemocytes were counted under the light microscope, following the Bürker double ruling.

### Bacterial strains and culture conditions

*Enterococcus mundtii* strain HDYM-22^38^, *Serratia marcescens* strain WW4 (provided by D. Carminati, CREA-FLC, Lodi, Italy) and *Micrococcus luteus* ATCC 4698 were cultured at 30 °C in Nutrient Broth (NB) or in Plate Count Agar (PCA) media.

### Silkworm oral infection, survival analysis and sample collection

For each *B*. *mori* strain, infection experiments were performed on fifth instar larvae derived from at least 10–15 egg-layings, reared in germ-free conditions. Single *E*. *mundtii* or *S*. *marcescens* colonies were grown overnight (ON) in NB medium. 2 mL of each bacterial suspension (in 0.9% NaCl; *E*. *mundtii*: 2.7 ∗ 10^2^ CFU/mL; *S*. *marcescens*: 2.23 ∗ 10^7^ CFU/mL) were homogeneously spread on the artificial diet. At the beginning of the light period, larvae were fed with the bacteria-supplemented diet for 24 h and transferred on a new germ-free diet. Control animals were fed in parallel for 24 h with a germ-free diet containing 2 mL 0.9% NaCl. For survival analysis, 3–6 replicates (15 silkworms each) per strain per condition were tested, recording daily the number of dead individuals.

All biological samples (hemolymph, midgut, fat bodies) were obtained collecting specimens from infected and uninfected larvae every 24 h during the seven days of the fifth instar. For each time-point, three-four replicates (each composed by tissues from three larvae) were collected under a laminar flow hood. Before dissection, silkworms were sequentially dipped in 70% ethanol, 5% sodium hypochlorite, and 70% ethanol.

### Isolation of bacteria from hemolymph

Hemolymph was collected as described above. For each pool, we plated on PCA 100 µL hemolymph, corresponding to the maximum volume collected from three larvae at the beginning of the fifth instar. Plates were incubated at room temperature for at least two days for CFU counts.

### Analysis of plasma humoral components

For antimicrobial and lysozyme activity determinations, hemolymph samples were mixed with 2.5 mM phenylthiourea to prevent melanization. After centrifugation (1000 × g, 5 min at 4 °C), hemocyte-free plasma was snap-frozen in liquid nitrogen. Before tests, the absence of living bacteria was checked on PCA plates.

#### Antimicrobial activity

*E*. *mundtii* and *S*. *marcescen*s cultures were diluted to 0.5 ∗ 10^5^ CFU/mL in PCA and Muller Hinton broth (Sigma) media, respectively. 25 µL of plasma were assayed against 200 µL of bacterial cultures on sterile polystyrene 96-well plates. As controls, both bacterial suspensions in growing medium (positive control) or with 50 µg/mL Ampicillin (negative control) were followed in parallel. Plates were incubated at 30 °C, 50 rpm. OD_600_ was measured at different time-points (0, 3, 5, 7, 9, 24 h). The bacterial growth rate (µ) was calculated using the formula: $\mu =2.303\times ({\rm{lg}}\,{{\rm{OD}}}_{tn}-\,{\rm{lg}}\,{{\rm{OD}}}_{to})/({{\rm{t}}}_{n}-{{\rm{t}}}_{0})$, with t_n_ indicating the different time-points and t_0_ the time 0, as in ref. 59. The area under each µ curve was calculated between 3 and 9 h *post*-*inoculum*, when both bacteria showed the highest growing rates in standard culture media.

#### Melanization response

Melanization of 100 µL plasma samples was measured reading the OD_450_ every 10 min for 50 min. As negative control, 2.5 mM phenylthiourea-supplemented plasma was used. ∆OD_450_ was calculated subtracting the OD_450_ recorded at time 0 to those obtained at the different time-points. To compare the melanization rate among plasma samples, linear regression was performed for each ∆OD_450_ versus time^60^.

#### Lysozyme activity

A single *M*. *luteus* colony was inoculated in NB at 30 °C ON. Bacteria were diluted in Reaction Buffer (66 mM Potassium Phosphate, pH 6.24) to a final OD_450_ of 0.6. 25 µL of plasma were tested in triplicate against 200 µL of *M*. *luteus* suspension on sterile polystyrene 96-well plates. 200 µL of *M*. *luteus* suspension were supplemented with a fresh lysozyme solution (200 Units/mL; positive control) or 25 µL Reaction Buffer (negative control). The OD_450_ was measured at time 0 and after 5 min. The lysozyme concentration was calculated using the formula: ${\rm{U}}=[-\frac{{\rm{\Delta }}\mathrm{OD}450}{5\,\,{\rm{\min }}}]/(0.001\ast 0.025\,\,{\rm{mL}})\,$, as in ref. 61.

### RNA extraction and qPCR analysis

Midgut and fat body samples were placed in RNA later solution and stored at −20 °C until RNA extraction. Total RNA was extracted using Trizol (Thermo Fisher Scientific) following the manufacturer’s protocol. Each qPCR reaction was performed in duplicate using Power SYBR Green RNA-to-CT 1-Step Kit (Thermo Fisher Scientific) in a 10 µL reaction volume, containing 200 nM of specific primers (Supplementary Table S10) and 20 ng RNA. For *att*, *cec A* and *B*, primers were designed to amplify all the elements of the multigene families (two for *att* and *cec A*; six for *cec B*). The amplification protocol included 30 min at 48 °C, 10 min at 95 °C and 40 cycles of 15 sec at 95 °C and 60 sec at 60 °C. The relative mRNA levels of AMP genes in uninfected conditions were determined calculating the 2^−∆Ct^ values of each AMP gene with respect to *actin3* using the formula: ${2}^{-{\rm{\Delta }}\mathrm{Ct}}={2}^{-({{\rm{Ct}}}_{AMP}-{{\rm{Ct}}}_{actin3})}$ and normalising these values to the highest 2^−∆Ct^ at the first day of the fifth larval stage (time 0) among the strains^62^.

For each strain, the daily relative contribution of the analysed AMP transcripts was calculated nomalising the 2^−∆Ct^ values of each AMP gene to the highest 2^−∆Ct^ value of *attacin* detected at the same day. Data were then expressed as percentages. Primer amplification efficiencies (E) were calculated from the slopes of standard curves using the formula: ${\rm{E}}={10}^{(-\frac{1}{slope})}$. Gene transcriptional variations during infections were determined as expression ratio between infected and uninfected samples using the formula: ${\rm{R}}=\frac{{\rm{E}}\,{{\rm{target}}}^{{\rm{\Delta }}\mathrm{Ct}{\rm{target}}({\rm{uninfected}}-{\rm{infected}})}}{{\rm{E}}\,{\rm{actin}}{3}^{{\rm{\Delta }}\mathrm{Ct}actin3({\rm{uninfected}}-{\rm{infected}})}}$ ^63^.

### AMP coding sequence amplification

AMP sequences were amplified from cDNAs, except for *cec A* and *B* genes, which were obtained from genomic DNAs. cDNAs were retrotranscribed from 2.5 µg of total RNA. Genomic DNA was extracted from midguts with a standard phenol:chloroform protocol. AMP sequences were obtained from at least 10 individuals per strain, except for *cec B6* in the European strain (n = 15), *glv 1* in European, Chinese and Japanese populations (n = 65) and *glv 2* in the European and Chinese strains (n = 40). PCRs were performed with 50 ng DNA, 0.2 U of a high-fidelity DNA Polymerase, 200 µM dNTPs and 300 nM of specific primers (Supplementary Table S10). The PCR cycle included 3 min at 98 °C, and 30 cycles of 98 °C for 1 min, 58–63 °C for 45 sec, 72 °C for 15 sec–2 min, depending on the amplicon length. Sanger sequences were analysed with CLC Sequence Viewer.

### *In silico* analysis of AMP isoforms

AMP active portions were *in silico* analysed with AMPA, CAMP, and APD3 tools^45–47^, evaluating the following parameters: net charge; Boman index (it represents the protein-binding potential, with hydrophobic peptides showing negative indices, and hydrophilic peptide tending to have positive indices); aliphatic index (the relative volume occupied by aliphatic side chains with high values indicating an increased thermostability); instability index (numerical values <40 for stable peptides); hydropathy (positive values indicate hydrophobicity).

### MIC assay

Cec B6 iso 1 and 2, carrying a C-terminal amidation^64^, were chemically synthesised with a purity ≥97% at Genescript; USA, resuspended in ultrapure water. Concentrations were determined at 280 nm. Serial dilutions (6, 3, 2.2, 1.8, 1.2, 0.8, 0.4, 0.2 and 0.1 µM) were tested in triplicate against 200 µL *E*. *mundtii* exponentially growing cultures (5 * 10^5^ CFU/mL initial concentration) on sterile polypropylene 96-well plates. Bacterial suspensions in growing medium (positive control) or with 50 µg/mL Ampicillin (negative control) were followed in parallel. Plates were incubated at 30 °C, 50 rpm. ∆OD_600s_ were calculated subtracting the OD_600_ recorded at time 0 to those obtained at the different time-points (3, 5, 7, 9 and 24 h). The experiment was performed twice.

### G157C SNP detection in *cec B* sequences of thirty-six non-assembled silkworm genomes

For each non-assembled genome obtained by ref. 29, the *cec B* sequences were downloaded from NCBI after a blastn search v 2.6.0^65^, using the entire genomic sequence of each *cec B* paralogue as query (BGIBMGA000021, BGIBMGA000023, BGIBMGA000024, BGIBMGA000036, BGIBMGA000037, BGIBMGA000038) and the strain-specific set of non-assembled sequences as database (accession number: SRA009208in the NCBI Short Read archive database). A 14 bp portion containing the G157C SNP was used as query in a blastn search against the *cec B* sequences of each strain. Alignments of retrieved sequences are available upon request from O.R.

### Statistical analysis

Survival analyses were performed using the Mantel-Cox test. Hemocyte numbers were compared using one-way ANOVA, followed by Newman-Keuls *post hoc* test. QPCR data on AMP expression did not approximate normal distributions, evaluated with Lilliefors (Kolmogorov-Smirnov) and Shapiro-Wilk tests. Therefore, they were non-parametrically analysed using Kruskal-Wallis test, followed by Dunn’s *post hoc* test. Plasma antimicrobial activity, melanization response, and lysozyme activity of infected and uninfected individuals from the four strains were compared using the two-way ANOVA, followed by Bonferroni *post hoc* test. The role of the *glv 1* alleles during infection was evaluated as number of dead or alive individuals carrying a specific allele using a 2X2 Contingency Table followed by Fisher exact test, and performing a logistic regression analysis in which the probability of the categorical dependent variable (dead or alive) was estimated with respect to the independent variable (genotypes), considered as factor. Analyses were performed using GraphPad Prism 6.07 or SPSS.

## Electronic supplementary material

Romoli et al_Supplementary Material
