# Supplementary material for: Differential sensitivity to infections and antimicrobial peptide-mediated immune response in four silkworm strains with different geographical origin
Source: Sci Rep. 2017 Apr 21;7:1048. doi: 10.1038/s41598-017-01162-z (PMC5430696; doi:10.1038/s41598-017-01162-z)
Supplement: Supplementary file 1 — Romoli et al_Supplementary Material [file 41598_2017_1162_MOESM1_ESM.pdf]

Differential sensitivity to infections and antimicrobial peptide-mediated immune response in four silkworm strains with different geographical origin

Supplementary material

Ottavia Romoli[1], Alessio Saviane[2], Andrea Bozzato[1], Paola D'Antona[3], Gianluca Tettamanti[3], Andrea Squartini[4], Silvia Cappellozza[2] and Federica Sandrelli[1]

[1] Department of Biology, University of Padova, Padova, Italy

[2] CREA - Honey Bee and Silkworm Research Unit, Padova Seat, Padova, Italy.

[3] Department of Biotechnology and Life Sciences, University of Insubria, Varese, Italy.

[4] Department of Agronomy, Food, Natural Resources, Animals and Environment, University of Padova, Padova, Italy

Supplementary Table S1. Statistical analysis of variations in AMP expression profiles in uninfected conditions and in AMP induction during *E. mundtii* and *S. marcescens* infections in the four silkworm strains

AMP gene	Tissue	Strain	Uninfected conditions				*E. mundtii* infection				*S. marcescens* infection			
			Kruskal-Wallis test			*post-hoc* test #	Kruskal-Wallis test			*post-hoc* test #	Kruskal-Wallis test			*post-hoc* test #
			value	p	S	(day 0 vs day n)	value	p	S	(day 0 vs day n)	value	p	S	(day 0 vs day n)
attacin	MG	I	1.769	0.67	ns	nd	9.80	0.0006	s	day 1	9.08	0.0006	s	day 1
		J	3.513	0.34	ns	nd	9.59	0.001	s	day 2	9.57	0.001	s	day 1
		C	3.359	0.37	ns	nd	8.56	0.01	s	day 1	8.55	0.01	s	day 1
		E	4.744	0.21	ns	nd	9.60	0.001	s	day 3	9.59	0.0013	s	day 3
	FB	I	5.333	0.50	ns	nd	17.99	0.006	s	day 4	18.09	0.006	s	day 1
		J	4.364	0.68	ns	nd	18.89	0.004	s	day 3	19.38	0.004	s	day 6
		C	11.8	0.07	ns	nd	13.00	0.04	s	day 4	4.21	0.65	ns	nd
		E	9.489	0.15	ns	nd	15.33	0.02	s	day 6	16.34	0.01	s	day 5
cecropin A	MG	I	1.154	0.80	ns	nd	8.87	0.006	s	day 2	8.86	0.006	s	day 2
		J	7.610	0.02	s	day 3	9.59	0.001	s	day 1	9.59	0.001	s	day 1
		C	1.769	0.67	ns	nd	9.84	0.0006	s	day 3	9.80	0.0006	s	day 3
		E	5.61	0.13	ns	nd	10.53	< 0.0001	s	day 3	10.53	< 0.0001	s	day 3
	FB	I	7.88	0.25	ns	nd	18.23	0.006	s	day 4	16.35	0.012	s	day 2
		J	11.29	0.08	ns	nd	13.70	0.03	s	day 2	19.44	0.004	s	day 6
		C	13.53	0.03	s	day 6	14.27	0.03	s	day 3	14.21	0.03	s	day 3
		E	3.29	0.77	ns	nd	16.84	0.01	s	day 6	18.85	0.004	s	day 6
cecropin B	MG	I	0.846	0.87	ns	nd	9.8	0.0006	s	day 1	9.80	0.0006	s	day 1
		J	2.385	0.54	ns	nd	10.53	< 0.0001	s	day 3	10.53	< 0.0001	s	day 3
		C	1.154	0.80	ns	nd	8.55	0.01	s	day 2	8.55	0.01	s	day 2
		E	3.820	0.32	ns	nd	10.12	0.0002	s	day3	10.12	0.0002	s	day 3
	FB	I	7.671	0.26	ns	nd	18.11	0.006	s	day 4	15.14	0.02	s	day 2

AMP gene	Tissue	Strain	Uninfected conditions				*E. mundtii* infection				*S. marcescens* infection			
			Kruskal-Wallis test			*post-hoc* test #	Kruskal-Wallis test			*post-hoc* test #	Kruskal-Wallis test			*post-hoc* test #
			value	p	S	(day 0 vs day n)	value	p	S	(day 0 vs day n)	value	p	S	(day 0 vs day n)
		J	8.121	0.22	ns	nd	14.2	0.03	s	day 2	16.35	0.012	s	day 6
		C	16.37	0.01	s	day 6	12.57	0.051	ns	nd	13.92	0.03	s	day 5
		E	7.72	0.26	ns	nd	16.96	0.01	s	day 5	18.82	0.005	s	day 5
cecropin D	MG	I	2.897	0.45	ns	nd	10.53	< 0.0001	s	day 1	10.53	< 0.0001	s	day 1
		J	3.103	0.42	ns	nd	8.35	0.01	s	day 2	8.34	0.01	s	day 2
		C	2.795	0.46	ns	nd	9.59	0.001	s	day 3	9.59	0.001	s	day 3
		E	4.744	0.21	ns	nd	9.49	0.002	s	day 1	9.49	0.002	s	day 1
	FB	I	13.52	0.03	s	day 6	17.19	0.01	s	day 4	15.24	0.02	s	day 2
		J	15.74	0.01	s	day 6	16.35	0.01	s	day 3	17.27	0.01	s	day 3
		C	14.12	0.03	s	day 6	14.44	0.03	s	day 5	18.82	0.005	s	day 4
		E	8.048	0.23	ns	nd	17.76	0.007	s	day 6	19.03	0.004	s	day 5
cecropin E	MG	I	4.846	0.19	ns	nd	7.30	0.04	s	day 1	7.30	0.04	s	day 1
		J	3.769	0.32	ns	nd	9.59	0.001	s	day 1	9.59	0.001	s	day 1
		C	2.59	0.51	ns	nd	7.72	0.02	s	day 2	9.72	0.02	s	day 2
		E	2.985	0.44	ns	nd	9.65	0.001	s	day 1	9.65	0.0007	s	day 1
	FB	I	7.654	0.26	ns	nd	19.1	0.004	s	day 4	15.9	0.01	s	day 2
		J	6.974	0.32	ns	nd	17.73	0.007	s	day 3	15.23	0.02	s	day 6
		C	4.71	0.58	ns	nd	16.41	0.01	s	day 4	16.94	0.01	s	day 3
		E	6.981	0.32	ns	nd	16.28	0.01	s	day 6	18.51	0.005	s	day 5
defensin A	MG	I	0.128	0.99	ns	nd	8.35	0.01	s	day 1	8.35	0.01	s	day 1
		J	1.462	0.74	ns	nd	10.53	< 0.0001	s	day 2	10.53	< 0.0001	s	day 2
		C	1.872	0.65	ns	nd	10.12	0.0002	s	day 1	10.13	0.0002	s	day 1
		E	3.348	0.39	ns	nd	9.60	0.001	s	day 3	9.59	0.001	s	day 3
	FB	I	8.104	0.23	ns	nd	16.53	0.01	s	day 4	18.26	0.006	s	day 5

AMP gene	Tissue	Strain	Uninfected conditions				*E. mundtii* infection				*S. marcescens* infection			
			Kruskal-Wallis test			*post-hoc* test #	Kruskal-Wallis test			*post-hoc* test #	Kruskal-Wallis test			*post-hoc* test #
			value	p	S	(day 0 vs day n)	value	p	S	(day 0 vs day n)	value	p	S	(day 0 vs day n)
		J	3.792	0.70	ns	nd	16.58	0.01	s	day 5	14.10	0.03	s	day 5
		C	0.675	0.99	ns	nd	14.72	0.02	s	day 4	16.42	0.01	s	day 6
		E	2.857	0.82	ns	nd	18.68	0.005	s	day 5	17.34	0.01	s	day 5
defensin B	MG	I	0.436	0.95	ns	nd	10.12	0.0002	s	day 3	9.49	0.002	s	day 2
		J	4.545	0.23	ns	nd	8.19	0.01	s	day 3	9.59	0.001	s	day 1
		C	0.128	0.99	ns	nd	8.19	0.01	s	day 2	8.55	0.01	s	day 2
		E	0.60	0.91	ns	nd	5.85	0.11	ns	nd	10.12	0.0002	s	day 2
	FB	I	9.822	0.13	ns	nd	19.38	0.004	s	day 4	14.88	0.02	s	day 1
		J	10.14	0.11	ns	nd	18.96	0.004	s	day 3	18.3	0.005	s	day 6
		C	7.815	0.25	ns	nd	18.89	0.004	s	day 4	18.51	0.005	s	day 2
		E	4.54	0.60	ns	nd	17.53	0.007	s	day 5	17.10	0.009	s	day 5
gloverin 2	MG	I	2.282	0.57	ns	nd	9.59	0.001	s	day 1	9.59	0.001	s	day 1
		J	2.692	0.48	ns	nd	10.53	< 0.0001	s	day 3	10.53	< 0.0001	s	day 3
		C	5.821	0.11	ns	nd	8.35	0.01	s	day 2	8.34	0.01	s	day 2
		E	2.179	0.58	ns	nd	10.12	0.0002	s	day 3	10.12	0.0002	s	day 3
	FB	I	7.827	0.25	ns	nd	15.90	0.01	s	day 5	14.13	0.03	s	day 4
		J	12.28	0.06	ns	nd	15.45	0.02	s	day 2	18.77	0.005	s	day 6
		C	9.786	0.13	ns	nd	15.35	0.02	s	day 4	17.99	0.006	s	day 5
		E	9.16	0.16	ns	nd	17.47	0.007	s	day 4	18.56	0.005	s	day 6
lebocin	MG	I	2.692	0.49	ns	nd	10.12	0.0002	s	day 1	10.12	0.0002	s	day 1
		J	1.769	0.67	ns	nd	7.41	0.03	s	day 2	7.41	0.03	s	day 2
		C	5.0	0.18	ns	nd	9.59	0.001	s	day 3	9.59	0.001	s	day 3
		E	2.59	0.51	ns	nd	9.59	0.001	s	day 1	9.59	0.001	s	day 1
	FB	I	9.039	0.17	ns	nd	18.30	0.005	s	day 3	17.67	0.0071	s	day 2

AMP gene	Tissue	Strain	Uninfected conditions				*E. mundtii* infection				*S. marcescens* infection			
			Kruskal-Wallis test			*post-hoc* test #	Kruskal-Wallis test			*post-hoc* test #	Kruskal-Wallis test			*post-hoc* test #
			value	p	S	(day 0 vs day n)	value	p	S	(day 0 vs day n)	value	p	S	(day 0 vs day n)
		J	15.58	0.01	s	nd	15.28	0.02	s	day 2	17.81	0.0067	s	day 4
		C	12.71	0.04	s	day 6	16.94	0.01	s	day 5	10.59	0.10	ns	nd
		E	11.98	0.06	ns	nd	16.46	0.01	s	day 6	19.13	0.004	s	day 6
moricin	MG	I	4.333	0.25	ns	nd	7.62	0.03	s	day 3	7.61	0.03	s	day 3
		J	4.015	0.29	ns	nd	10.12	0.0002	s	day 3	10.12	0.0002	s	day 3
		C	7.308	0.03	s	day 2	10.53	< 0.0001	s	day 3	10.53	< 0.0001	s	day 3
		E	4.846	0.19	ns	nd	9.8	0.0006	s	day 1	9.80	0.0006	s	day 1
	FB	I	8.554	0.20	ns	nd	18.68	0.005	s	day 4	17.29	0.008	s	day 2
		J	11.50	0.07	ns	nd	14.34	0.005	s	day 3	19.38	0.004	s	day 4
		C	11.32	0.07	ns	nd	15.49	0.03	s	day 5	10.59	0.10	ns	nd
		E	6.286	0.39	ns	nd	16.94	0.01	s	day 3	19.03	0.0041	s	day 5

S: significance with "s" significant and "ns" not significant.
post-hoc test (day 0 vs day n): the first day in which expression data resulted significantly different from those collected at the beginning of larval development in uninfected conditions (day 0). Results obtained with Dunn's *post-hoc* test.
I: Indian; J: Japanese; C: Chinese; E: European.

Supplementary Table S2. Statistical analysis of *E. mundtii* growth rates in plasma of the four *B. mori* strains collected during six days after infection

**Comparison**	**two-way ANOVA**					**Bonferroni *post-hoc* test (p<0.05)**					
	**Source of variation**		**F**	**p**	**S**	**Day 1**	**Day 2**	**Day 3**	**Day 4**	**Day 5**	**Day 6**
**Inter-strain comparison among uninfected strains**	strain		F (3, 48) = 13.50	p < 0.0001	s	E>I,C,J	E>C	ns	ns	ns	ns
	time		F (5, 48) = 1.735	p = 0.1447	ns						
	time X strain		F(15,48) = 1.53	p = 0.13	ns						
**Intra-strain comparison infected *vs* uninfected**	I	condition	F (1, 24) = 114.6	p < 0.0001	s	ns	uninf > inf	uninf > inf	uninf > inf	uninf > inf	uninf > inf
		time	F (5, 24) = 0.1386	p = 0.9816	ns						
		time X condition	F (5, 24) = 1.094	p = 0.3894	ns						
	C	condition	F (1, 24) = 797.4	p < 0.0001	s	uninf > inf	uninf > inf	uninf > inf	uninf > inf	uninf > inf	uninf > inf
		time	F (5, 24) = 22.69	p < 0.0001	s						
		time X condition	F (5, 24) = 17.17	p < 0.0001	s						
	J	condition	F (1, 24) = 447.2	p < 0.0001	s	ns	uninf > inf	uninf > inf	uninf > inf	uninf > inf	uninf > inf
		time	F (5, 24) = 6.798	p = 0.0004	s						
		time X condition	F (5, 24) = 14.55	p < 0.0001	s						
	E	condition	F (1, 24) = 476.3	p < 0.0001	s	uninf > inf	uninf > inf	uninf > inf	uninf > inf	uninf > inf	uninf > inf
		time	F (5, 24) = 3.790	p = 0.0114	s						
		time X condition	F (5, 24) = 1.504	p = 0.2258	ns						
**Inter-strain comparison among infected strains (excluding day 1)**	strain		F (3, 40) = 33.06	p < 0.0001	s	-	J>E	J>E	J,I>E	J,I>E	I,C,J>E
	time		F (4, 40) = 39.18	p < 0.0001	s						
	time X strain		F (12, 40) = 22.51	p < 0.0001	s						

S: significance with "s" significant and "ns" not significant.
I: Indian; C: Chinese; J: Japanese; E: European.

Supplementary Table S3. Statistical analysis of *S. marcescens* growth rates in plasma of the four *B. mori* strains collected during six days after infection

Comparison		two-way ANOVA				Bonferroni *post-hoc* test (p<0.05)					
		Source of variation	F	p	S	Day 1	Day 2	Day 3	Day 4	Day 5	Day 6
Inter-strain comparison among uninfected strains		strain	F (3, 48) = 3.973	p = 0.0131	s	E>J	C>J	ns	J>E,I	ns	ns
		time	F (5, 48) = 7.832	p < 0.0001	s						
		time X strain	F (15, 48) = 2.648	p = 0.0054	s						
Intra-strain comparison infected *vs* uninfected	I	condition	F (1, 24) = 101.9	p < 0.0001	s	uninf > inf	uninf > inf	uninf > inf	uninf > inf	uninf > inf	uninf > inf
		time	F (5, 24) = 0.6919	p = 0.6345	ns						
		time X condition	F (5, 24) = 1.253	p = 0.3160	ns						
	C	condition	F (1, 22) = 1.015	p = 0.3248	ns	ns	ns	ns	ns	ns	ns
		time	F (5, 22) = 1.774	p = 0.1599	ns						
		time X condition	F (5, 22) = 1.745	p = 0.1662	ns						
	J	condition	F (1, 23) = 2.192	p = 0.1523	ns	ns	ns	ns	ns	ns	ns
		time	F (5, 23) = 4.051	p = 0.0088	s						
		time X condition	F (5, 23) = 1.586	p = 0.2036	ns						
	E	condition	F (1, 24) = 10.98	p = 0.0029	s	uninf > inf	ns	ns	ns	ns	ns
		time	F (5, 24) = 0.7360	p = 0.6038	ns						
		time X condition	F (5, 24) = 2.326	p = 0.0740	ns						

S: significance with "s" significant and "ns" not significant.
I: Indian; C: Chinese; J: Japanese; E: European.

Supplementary Table S4. Statistical analysis of melanization rates in *E. mundtii*-infected and uninfected samples of the four *B. mori* strains

**two-way ANOVA**					**Bonferroni *post-hoc* test (p<0.05)**						
**Source of variation**		**F**	**p**	**S**	**Day 0**	**Day 1**	**Day 2**	**Day 3**	**Day 4**	**Day 5**	**Day 6**
I	condition	$F (1, 51) = 1054$	$p < 0.0001$	s	ns	uninf < inf	uninf < inf	uninf > inf	uninf > inf	uninf > inf	nd
	time	$F (6, 51) = 66.15$	$p < 0.0001$	s							
	time X condition	$F (6, 51) = 299.9$	$p < 0.0001$	s							
C	condition	$F (1, 130) = 1745$	$p < 0.0001$	s	ns	uninf > inf	uninf > inf	uninf > inf	uninf > inf	uninf > inf	uninf > inf
	time	$F (6, 130) = 1185$	$p < 0.0001$	s							
	time X condition	$F (6, 130) = 162.9$	$p < 0.0001$	s							
J	condition	$F (1, 28) = 1152$	$p < 0.0001$	s	ns	uninf > inf	uninf > inf	uninf > inf	uninf > inf	uninf > inf	uninf > inf
	time	$F (6, 28) = 380.6$	$p < 0.0001$	s							
	time X condition	$F (6, 28) = 84.59$	$p < 0.0001$	s							
E	condition	$F (1, 28) = 457$	$p < 0.0001$	s	ns	uninf < inf	uninf < inf	uninf > inf	uninf > inf	uninf > inf	uninf > inf
	time	$F (6, 28) = 440$	$p < 0.0001$	s							
	time X condition	$F (6, 28) = 152.2$	$p < 0.0001$	s							

S: significance with "s" significant and "ns" not significant.
nd: not determined.
I: Indian; C: Chinese; J: Japanese; E: European.

Supplementary Table S5. Statistical analysis of melanization rates in *S. marcescens*-infected and uninfected samples of the four *B. mori* strains

two-way ANOVA					Bonferroni *post-hoc* test (p<0.05)						
Source of variation		F	p	S	Day 0	Day 1	Day 2	Day 3	Day 4	Day 5	Day 6
I	condition	F (1, 51) = 0.6138	p = 0.4370	ns	ns	uninf < inf	uninf < inf	uninf < inf	uninf > inf	uninf > inf	ns
	time	F (6, 51) = 119.0	p < 0.0001	s							
	time X condition	F (6, 51) = 62.48	p < 0.0001	s							
C	condition	F (1, 130) = 70.07	p < 0.0001	s	ns	uninf > inf	ns	uninf < inf	uninf > inf	uninf > inf	uninf < inf
	time	F (6, 130) = 1334	p < 0.0001	s							
	time X condition	F (6, 130) = 127.2	p < 0.0001	s							
J	condition	F (1, 28) = 208.6	p < 0.0001	s	ns	uninf > inf	ns	uninf > inf	uninf > inf	ns	uninf > inf
	time	F (6, 28) = 187.1	p < 0.0001	s							
	time X condition	F (6, 28) = 20.96	p < 0.0001	s							
E	condition	F (1, 46) = 240.6	p < 0.0001	s	ns	uninf > inf	uninf > inf	uninf > inf	uninf > inf	uninf > inf	ns
	time	F (6, 46) = 180.1	p < 0.0001	s							
	time X condition	F (6, 46) = 23.38	p < 0.0001	s							

S: significance with "s" significant and "ns" not significant.
nd: not determined.
I: Indian; C: Chinese; J: Japanese; E: European.

Supplementary Table S6. Plasma lysozyme activity in *E. mundtii* and *S. marcescens* infected silkworms and uninfected controls

Strain	Condition	Day 1	Day 2	Day 3	Day 4	Day 5	Day 6	p
Indian	uninfected	65.9 ± 25.9	64.8 ± 8.9	64.8 ± 25.5	59.2 ± 21.9	56.8 ± 9.8	24 ± 2.6	
	E. mundtii	16.7 ± 8.4	14.5 ± 14.5	25.4 ± 25.4	37.3 ± 37.3	35.5	0 ± 0.0	<0.05
	S. marcescens	37.6 ± 31.0	8.2 ± 4.1	50.4 ± 22.2	6.9 ± 6.9	10.9 ± 9.4	34.1 ± 6.4	<0.05
Chinese	uninfected	40.5 ± 8.8	37.1 ± 5.6	45.7 ± 25.4	31.7 ± 3.2	41.0 ± 2.7	2.0 ± 2.0	
	E. mundtii	50.1 ± 8.8	0 ± 0.0	0 ± 0.0	3.3 ± 3.3	0 ± 0.0	0 ± 0.0	<0.001
	S. marcescens	51.7 ± 7.2	28.3 ± 2.9	42.4 ± 12.0	25.1 ± 10.4	48.3 ± 8.1	37.9 ± 11.6	0.35, ns
Japanese	uninfected	33.3 ± 0.8	25.2 ± 13.1	58.1 ± 12.0	36.9 ± 18.8	56.8 ± 12.0	0 ± 0.0	
	E. mundtii	85.1 ± 46.4	21.1 ± 21.1	5.9 ± 5.9	17.1 ± 17.1	0 ± 0.0	5.1 ± 5.1	0.24, ns
	S. marcescens	50.7 ± 1.9	52.5 ± 7.0	45.3 ± 26.2	52.3 ± 6.2	48.8 ± 12.1	58.1 ± 13.9	<0.05
European	uninfected	56.0 ± 11.3	80.3 ± 23.8	24.3 ± 15.3	24.0 ± 7.9	33.4 ± 22.0	23.5 ± 20.5	
	E. mundtii	96.8 ± 42.6	12.7 ± 7.9	71.9 ± 52.4	19.5 ± 2.5	17.9 ± 17.9	71.1 ± 49.0	0.62, ns
	S. marcescens	26.4 ± 13.6	33.1 ± 15.5	19.7 ± 5.9	47.7 ± 11.3	50.9 ± 40.2	33.6 ± 6.7	0.64, ns

Lysozyme activity is expressed in Units/mL (mean ± SEM; nine plasma in three replicates per time per condition); P: two-way ANOVA between infected and uninfected samples within the same strain (effect of infection): *E. mundtii*: Indian: $F_{1,22} = 12.01$, p = 0.002; Chinese: $F_{1,23} = 20.3$, p = 0.0002; Japanese: $F_{1,23} = 1.475$, p = 0.24; European: $F_{1,24} = 0.253$, p = 0.62; *S. marcescens*: Indian: $F_{1,24} = 14.08$, p = 0.001; Chinese: $F_{1,22} = 0.913$, p = 0.35; Japanese: $F_{1,23} = 4.324$, p = 0.048; European: $F_{1,24} = 0.223$, p = 0.64; ns: not significant.

Supplementary Table S7. Polymorphic positions in AMP genes and peptides of the four *B. mori* strains

Gene (cds length)	Alleles	SNPs	Strains				AMP Isoforms	aa substitution	Frequency (%s)			
			I	C	J	E			I	C	J	E
att 1 (645 bp)	ref. sequence BGIBMGA002747	105G 108T 162A 234G 240T **241A** 261C 321C 333T 378G 390T 471C 513G 537T					1	-	100	100	100	100
	1	105G 108T 162A 234G 240T **241G** 261C 321C 333T 378G 390T 471C 513G 537T	+									
	2	105G 108C 162G 234A 240C **241G** 261T 321C 333T 378T 390C 471G 513C 537C		+	+							
	3	105A 108C 162G 234G 240T **241G** 261C 321T 333C 378G 390T 471G 513G 537T			+	+						
att 2 (645 bp)	ref. sequence BGIBMGA002739	108T 201C 219C 240T **241A** 498T 627C	+				1	V81	100			
	2	108C 201T 219T 240C **241G** 498C 627T		+	+	+	2	I81		100	100	100
cec A1 (192 bp)	ref. sequence BGIBMGA006280	-	+	+	+	+	1	-	100	100	100	100
cec A2 (192 bp)	ref. sequence BGIBMGA014285	-	+	+	+	+	1	-	100	100	100	100
cec B1 (192 bp)	ref. sequence BGIBMGA000024	-	+	+	+	+	1	-	100	100	100	100
cec B2 (192 bp)	ref. sequence BGIBMGA000023	-	+	+	+	+	1	-	100	100	100	100
cec B3 (192 bp)	ref. sequence BGIBMGA000021	**112G**		+	+	+	1	G38		100	100	100
	2	**112A**	+				2	S38	100			
cec B4 (192 bp)	ref. sequence BGIBMGA000036	-	+	+	+	+	1	-	100	100	100	100
cec B5 (192 bp)	ref. sequence BGIBMGA000037	-	+	+	+	+	1	-	100	100	100	100

Gene (cds length)	Alleles	SNPs	Strains				AMP Isoforms	aa substitution	Frequency (%s)			
			I	C	J	E			I	C	J	E
cec B6 (192 bp)	ref. sequence BGIBMGA000038	123C **157G**	+				1	E53	100	100	100	
	2	123T **157G**		+	+							
	3	123T **157C**				+	2	Q53				100
cec D (186 bp)	ref. sequence BGIBMGA000017	154T	+				1	-				
	2	154C		+	+	+						
cec D2 (201 bp)	ref. sequence NM_001043459.1	**25G 76T** 99G 102A 117A 132C 153G 171T										
	1	**25A 76T** 99T 102G 117G 132C 153A 171C	+	+	+		1a	I9 F26	100	60	100	
	2	**25G 76T** 99T 102G 117G 132C 153A 171C		+			1b	V9 F26		40		
	3	**25G 76C** 99T 102G 117G 132T 153A 171C				+	1c	V9 L26				100
cec E (198 bp)	ref. sequence DQ233467.1	43T 108G 156C 165A 189T										
	1	43C 108G 156C 165A 189T	+	+			1	-				
	2	43T 108A 156T 165G 189C		+	+	+						
def A (279 bp)	ref. sequence NM_001043905	**130T**	+	+	+	+	1a	S44	100	100	100	40
	2	**130C**				+	1b	P44				60
def B (249 bp)	ref. sequence NM_001135205.1	**37G 40T** 45T 51G 81A **93G 110G 112C** 138A **181T**	+				1	V13 Y14 M31 R37 L38 Y61	100			

Gene (cds length)	Alleles	SNPs	Strains I	Strains C	Strains J	Strains E	AMP Isoforms	aa substitution	Frequency (%s) I	Frequency (%s) C	Frequency (%s) J	Frequency (%s) E
	2	**37A 40C** 45G 51G 81A **93A 110G 112A** 138A **181C**		+			2a	I13 H14 I31 R37 M38 H61		80		
	3	**37A 40C** 45G 51C 81G **93G 110G 112A** 138A **181C**		+			2b	I13 H14 M31 R37 M38 H61		20		
	4	**37A 40C** 45G 51G 81A **93G 110C 112C** 138G **181C**			+	+	2c	I13 H14 M31 T37 L38 H61			100	100
enb 1 (180 bp)	ref. sequence BGIBMGA000018	**73T** 96C 111A 114T 150A **152G** 153C **160G 163G** 174C	+	+		+	1	F25 G51 A54 A55	100	65		55
	2	**73A** 96T 111C 114C 150G **152C** 153G **160A 163T** 174T		+	+	+	2	I25 A51 T54 S55		35	100	45
enb 2 (180 bp)	ref. sequence BGIBMGA000039	9C 48G **73T** 96C 111A 114T 150A **152G** 153C **160G 163G** 174C	+	+		+	1	F25 G51 A54 A55	100	100		60
	2	9C 48A **73T** 96C 111A 114T 150A **152G** 153C **160G 163G** 174C		+								
	3	9T 48G **73A** 96T 111C 114C 150G **152C** 153G **160A 163T** 174T			+	+	2	I25 A51 T54 S55			100	40
glv 1 (537 bp)	ref. sequence BGIBMGA013863	27T **31C 47A 110A** 144T 150T **158A 169G** 219T 231C 255C 300G 309C 312C 348T 405A 459C	+		+		1a	L11 N16 K37 R40 K53 G57	100		100	
	2	27C **31C 47G 110G** 144C 150A **158A 169G** 219C 231C 255C 300A 309T 312C 348C 405C 459T		+			1b	L11 S16 R37 R40 K53 G57		59		42
	3	27T **31C 47G 110G** 144T 150T **158A 169G** 219T 231C 255C 300G 309C 312T 348T 405C 459C				+						
	4	27T **31T 47A 110G** 144T 150T **158G 169G** 219T 231T 255T 300A 309C 312C 348T 405C 459C		+			2	F11 N16 R37 R40 R53 G57		35		
	5	27T **31C 47G 110G** 144C 150T **158A 169A** 219T 231C 255C 300A 309C 312T 348T 405C 459C		+		+	3	L11 S16 R37 R40 K53 R57		6		58

Gene (cds length)	Alleles	SNPs	Strains				AMP Isoforms	aa substitution	Frequency (%s)			
			I	C	J	E			I	C	J	E
glv 2 (522 bp)	ref. sequence BGIBMGA005658	24C 51C **71C** 81A 90C **99C** 105A **106T** 123C 195C **221A** 270T 276G 342A 411G 492T 495T	+			+	1	S24 S33 S36 K74	100			40
	2	24A 51T **71T** 81A 90T **99G** 105G **106C** 123T 195A **221G** 270C 276A 342A 411G 492A 495C		+			2a	F24 R33 P36 R74		100		
	3	24C 51C **71C** 81G 90C **99C** 105A **106T** 123T 195A **221G** 270C 276A 342A 411G 492T 495T			+		2b	S24 S33 S36 R74			100	60
	4	24C 51C **71C** 81G 90C **99C** 105A **106T** 123T 195C **221G** 270T 276G 342T 411T 492T 495T				+						
glv 3 (519 bp)	ref. sequence BGIBMGA013803	**55G 70T** 78T **82A 92C** 93C **100G** 108T **146G** 198A 246T 249G 255C 276G 348T 387G 438C 489T 513T		+			1a	E19 S24 K28 P31 G34 R49		70		
	2	**55G 70C** 78C **82G 92T** 93T **100A** 108C **146G** 198C 246T 249G 255C 276A 348C 387G 438T 489C 513T	+			+	1b	E19 P24 E28 L31 S34 R49	100			45
	3	**55A 70C** 78C **82G 92C** 93C **100G** 108T **146G** 198C 246A 249G 255T 276G 348T 387G 438C 489C 513C			+		1c	K19 P24 E28 P31 G34 R49			70	
	4	**55G 70C** 78C **82G 92C** 93C **100G** 108T **146A** 198C 246T 249A 255C 276G 348C 387A 438C 489C 513T		+	+	+	2	E19 P24 E28 P31 G34 K49		30	30	55
glv 4 (516 bp)	ref. sequence BGIBMGA013865	**65C 70T 88A** 96T **99G** 102C 135T 147G 168T 180T 282C 396T **448A**										
	1	**65G 70G 88G** 96T **99G** 102T 135C 147G 168G 180T 282C 396T **448G**	+		+		1a	D24 V30 Q33	100		100	
	2	**65G 70T 88A** 96A **99T** 102C 135C 147A 168G 180C 282T 396C **448G**		+			1b	Y24 I30 H33		100		
	3	**65G 70T 88A** 96T **99T** 102T 135C 147A 168G 180C 282C 396T **448G**				+	1c	Y24 F30 H33				100
leb (540 bp)	ref. sequence BGIBMGA006775	**83A** 189T **320C 495T 499G** 525C	+		+	+	1a	Y28 T107 H165 N167	100		10	100
	2	**83T** 189C **320T 495A 499A** 525T		+	+		1b	F28 I107 Q165 N167		100	90	

Gene (cds length)	Alleles	SNPs	Strains				AMP Isoforms	aa substitution	Frequency (%s)			
			**I**	**C**	**J**	**E**			**I**	**C**	**J**	**E**
mor (201 bp)	ref. sequence BGIBMGA011495	-	+	+	+	+	1	-	100	100	100	100

cds: coding sequence.
SNP: single nucleotide polymorphism (position, nucleotide). Non synonymous substitution are indicated in bold.
Strains: I (Indian), C (Chinese), J (Japonese), E (European).
AMP Isoforms: peptide variants with the same active sequence, but with modifications in the pro-peptide or in the pre-pro-peptide, were indicated with different letters (e.g. 1a, 1b, etc.).
aa substitutions: amino acid modifications (amino acid, position).
Frequency (%s): frequencies of the isoforms in the four strains.
Underlined residues map in the active portion of the peptide.

Supplementary Table S8. Geographical origin of the *B. mori* and *B. mandarina* silkworms

Silkworm strain	Geographical origin, species
D01	Japan, *B. mori*
D02	Japan, *B. mori*
D03	Japan, *B. mori*
D04	Japan, *B. mori*
D05	Japan, *B. mori*
D06	Cambodia, *B. mori*
D07	Laos, *B. mori*
D08	India, *B. mori*
D09	Europe, *B. mori*
D10	Europe (Italy), *B. mori*
D11	Europe (Russia), *B. mori*
D16	China, *B. mori*
D17	China, *B. mori*
D18	China, *B. mori*
D19	China, *B. mori*
D20	China, *B. mori*
D21	China, *B. mori*
D22	China, *B. mori*
D23	China, *B. mori*
D24	China, *B. mori*
D25	China, *B. mori*
D26	China, *B. mori*
D27	China, *B. mori*
D28	China, *B. mori*
D29	China, *B. mori*
D_I	India (strain analysed in this study), *B. mori*
D_J	Japan (strain analysed in this study), *B. mori*
D_C	China (strain analysed in this study), *B. mori*
D_E	Europe (strain analysed in this study), *B. mori*
W01	Sichuan province, China, *B. mandarina*
W02	Sichuan province, China, *B. mandarina*
W03	Sichuan province, China, *B. mandarina*
W04	Sichuan province, China, *B. mandarina*
W05	Shanxi province, China, *B. mandarina*
W06	Hubei province, China, *B. mandarina*
W07	Jiangsu province, China, *B. mandarina*
W08	Sichuan province, China, *B. mandarina*
W09	Hunan province, China, *B. mandarina*
W10	Jiangsu province, China, *B. mandarina*
W11	Chongquin, China, *B. mandarina*

Modified from [29].

Supplementary Table S9. Statistical analysis of *Imd*, *Nos* and *Jhamt* expression induction in the four strains after *S. marcescens* infection

Gene	Tissue	two-way ANOVA				Bonferroni *post-hoc* test (p<0.05)	
		**Source of variation**	**F**	**p**	**S**	**Day 1**	**Day 2**
Imd	MG	strain	F (3,16) = 62.1	p < 0.0001	s	I > C, J, E	I > C, J, E
		time	F (1,16) = 0.4271	p = 0.5227	ns		
		time X strain	F (3,16) = 6.384	p = 0.0047	s		
	FB	strain	F (3,16) = 86.04	p < 0.0001	s	ns	I > C, J, E
		time	F (1,16) = 95.48	p < 0.0001	s		
		time X strain	F (3,16) = 62.27	p < 0.0001	s		
Nos	MG	strain	F (3,16) = 20.11	p < 0.0001	s	I > C, J, E	ns
		time	F (1,16) = 11.51	p = 0.0037	s		
		time X strain	F (3,16) = 21.98	p < 0.0001	s		
	FB	strain	F (3,16) = 27.54	p < 0.0001	s	I, C > J, E	I > C, J, E
		time	F (1,16) = 2.963	p = 0.1045	ns		
		time X strain	F (3,16) = 18.49	p < 0.0001	s		
Jhamt	MG	strain	F (3,16) = 29.12	p < 0.0001	s	I > C, J, E	I > C, J, E
		time	F (1,16) = 0.021	p = 0.8863	ns		
		time X strain	F (3,16) = 0.7098	p = 0.561	ns		
	FB	strain	F (3,16) = 1.980	p = 0.1576	ns	nd	nd
		time	F (1,16) = 0.153	p = 0.7009	ns		
		time X strain	F (3,16) = 2.807	p = 0.0731	ns		

S: significance with "s" significant and "ns" not significant.
nd: not determined.
I: Indian; C: Chinese; J: Japanese; E: European.

Supplementary Table S10. List of primers

Gene	Reference sequence	Experiment type	Primer name	Position	5'-3' sequence	Primer efficiency
actin 3	NM_001126254.1	qPCR	ACT3_qPCR_FOR	688-705	CGGGAAATCGTTCGTGAT	99.25%
			ACT3_qPCR_REV	851-868	ACGAGGGTTGGAAGAGGG	
attacin 1	NM_001043541.1	qPCR	ATT_qPCR_FOR	255-274	CAGGGCTCGCTCTGGACAAT	103.2%
			ATT_qPCR_REV	304-322	GAAGCCGGGAATGCGGGTC	
		Polymorphisms	ATT1_FOR	33-55	AGATGTCCAAGAGTGTAGCGTTG	
			ATT1_REV	794-822	GGTTCTTTATTTGATAAGGCAGTACTGAC	
attacin 2	BGIBMGA002739	Polymorphisms	ATT1_FOR	599-621	AGATGTCCAAGAGTGTAGCGTTG	
			ATT2_REV	1513-1540	CTAGAAATTCATTCGAGTCATGAAGCAG	
cecropin A1	BGIBMGA006280-RA	qPCR	CECA_qPCR_FOR	70-90	CCTGAGCCCAGGTGGAAACTC	93.07%
			CECA_qPCR_REV	143-162	GACGGCTATAGCTGGACCCG	
	scaf49:1257663-1259638	Polymorphisms	CECA1_FOR	383-407	TATGCATTCCATCGTATAGCAATTT	
			CECA1_REV	1388-1409	GCTAAATGCGTCTTTGGTGCTA	
cecropin A2	BGIBMGA014285	Polymorphisms	CECA2_FOR	390-413	TCCTCCATACAGCGTATTTGTGAC	
	scaf821:802-3114		CECA2_REV	1877-1901	GAAGGCAATGACTGTGGTATTCTTA	
cecropin B1	BGIBMGA000024-RA	qPCR	CECB_qPCR_FOR	31-51	TTCGCTCTGGTGCTGGCTTTG	94.92%
			CECB_qPCR_REV	130-148	GGCCCGCTTTGACGATGCC	
	scaf98:346932-349188	Polymorphisms	CECB1_FOR	460-479	CGTCTAACAACAAGCCACGC	
			CECB1_REV	1920-1944	TGTTGTTCTCTTAGTGTGTTGGTTC	
cecropin B2	BGIBMGA000023	Polymorphisms	CECB2_OUT_REV	scaffold 98	GACGCAAATGAGTCATTGTTGTTGG	
	scaf98:353687-355482		CECB2_FOR	317-341	GGTTGAGCTTTGTTAAAATGCGAGA	
			CECB2_REV	1203-1227	ACGAGTAGATTTATGGCGTACGTTT	
cecropin B3	BGIBMGA000021	Polymorphisms	CECB3_OUT_FOR	scaffold 98	TCAAAGCCCGCCTAGATGGC	
	scaf98:359256-361039		CECB3_OUT_REV	scaffold 98	GCAATGTCCGCGGTGGAGT	
			CECB3_FOR	163-182	TGTTAGCTCGTCCACCCAAC	
			CECB3_REV	1508-1534	CGTCACTGTAGATATTAAAAATGCGCT	
cecropin B4	BGIBMGA000036	Polymorphisms	CECB4_OUT_FOR	scaffold 98	AAGGGACAGTTTACTTCGCTTGC	
	scaf98:367515-369407		CECB4_OUT_REV	scaffold 98	GTTGATTGGCAGACTCCCTGC	

Gene	Reference sequence	Experiment type	Primer name	Position	5'-3' sequence	Primer efficiency
			CECB4_FOR	397-416	AGGGAAGTACCCCTCTCGTG	
			CECB4_REV	1512-1538	AGGTAGTAGTGGTTTAAGGATTGATTA	
cecropin B5	BGIBMGA000037	Polymorphisms	CECB5_OUT_FOR	scaffold 98	ATCCGGCGAGAAACTCAGC	
	scaf98:386831-388774		CECB5_OUT_REV	scaffold 98	GATCATTTTCCTATAGCTTTAGCCG	
			CECB5_FOR	390-407	CAGAGCGGGCGGTACTTC	
			CECB5_REV	1684-1706	CGTGAATGGTGACGTCAATGTAG	
cecropin B6	BGIBMGA000038	Polymorphisms	CECB6_OUT_REV	scaffold 98	AACTAAGTGTGAAAGTGTCGTCAC	
	scaf98:392963-394760		CECB6_FOR	62-81	CCAGTTTGGCTGTGAACTCC	
			CECB6_REV	1323-1347	TCACTAAGAAAACTTTGGCAGACAT	
cecropin D	BGIBMGA000017-RA	qPCR	CECD_qPCR_FOR	45-62	CGCCACGGCTTCGGTCTC	109.1%
			CECD_qPCR_REV	135-153	GGTGTCGACTGCTGGAGCC	
		Polymorphisms	CECD_FOR	1-28	ATGAAAATCTCGAAAATTTCGTTTTCG	
			CECD_REV	164-186	CTATCCTTGTCCGAGAGCTTTTG	
cecropin D2	NM_001043459.1	Polymorphisms	CECD2_FOR	2-19	AGGCCGTAGAGCCCACAA	
			CECD2_REV	238-262	CTCTGCTCTACTACGACACTCTATC	
cecropin E	DQ233467.1	qPCR	CECE_qPCR_FOR	110-129	GTGTGTGCGAGCGTTATGGC	108.5%
			CECE_qPCR_REV	238-256	CCCATGAGCGATGGTCGCC	
		Polymorphisms	CECE_FOR	33-57	GCCTCTCAACTAACGTATTCTTTGG	
			CECE_REV	315-338	AAGTCTTTCACAGTCTATTGAGCG	
defensin A	NM_001043905	qPCR	DEF_A_qPCR_FOR	130-148	CGTTGCCGAGAGACGCAAC	93.43%
			DEF_A_qPCR_REV	226-244	TCGCACCATATCCGCCCAG	
		Polymorphisms	DEF_A_FOR	13-39	TGAGCAAGTTTCTGTATTTCTAGTCTG	
			DEF_A_REV	346-375	TTTATTTGGCATTAACTAAAATGTATCCTT	
defensin B	NM_001135205.1	qPCR	DEF_B_qPCR_FOR	13-32	TGAGCCGTGTACGAGTGAAC	89.71%
			DEF_B_qPCR_REV	289-316	CCAACAGTATTGTTCTGATGAGAGATAG	
		Polymorphisms	DEF_B_FOR	13-32	TGAGCCGTGTACGAGTGAAC	
			DEF_B_REV	141-160	CTCCTGGATCGAAACAGCCT	
enbocin 1	NM_001044007.1	Polymorphisms	ENB1_FOR	1-20	GAGCTCGAACCCCGCTTTAG	
			ENB1_REV	270-293	AATGCTGTTCCAAATAGTTGTGCG	

Gene	Reference sequence	Experiment type	Primer name	Position	5'-3' sequence	Primer efficiency
enbocin 2	NM_001098374.1	Polymorphisms	ENB2_FOR	7-25	TGTTCCTGACTCGGCACTC	
			ENB2_REV	513-537	TGTCTCCAAAAGGCACTTTTATTGG	
gloverin 1	NM_001043465.1	Polymorphisms	GLV1_FOR	8-27	GTCTCGAGCAGCGAAACCTG	
			GLV1_REV	630-653	CATACTTCTAGGCTTACGAGGCAA	
	scaf62:1029376-1033274	Allele frequency	GLV1_freq_FOR	scaffold 62	GTCAGTCCGACACCCTCGC	
			GLV1_freq_REV	scaffold 62	CCCGATATTTTGTACTTACAAAAAGTCC	
gloverin 2	NM_001044218.2	qPCR	GLV2_qPCR_FOR	367-398	GCGAACAAGAATGCACAAGCCAC	101.3%
			GLV2_qPCR_REV	550-573	GTGTTGAGGTGATCACCAATCATG	
		Polymorphisms	GLV2_FOR	7-28	GGTCTTGAGGAGCGAAACTTGA	
			GLV2_REV	713-735	CGTCATAACAAAGCACGAGATCC	
gloverin 3	NM_001099842.1	Polymorphisms	GLV3_FOR	31-57	CAACTCAAAATGAATTCCAAATTGCTG	
			GLV3_REV	704-730	CAAGTTATCAGTAAGACATATTTCGTT	
gloverin 4	NM_001044219.1	Polymorphisms	GLV4_FOR	1-21	AGGAGCGAAACCTGTCACAAG	
			GLV4_REV	723-753	AGAGAAAGTGTAAAGTAGAATACTGTAGCTA	
Imd	BGIBMGA003655-RA	qPCR	IMD_qPCR_FOR	119-139	CGGTAGATGACGAACATGCAG	98.53%
			IMD_qPCR_FOR	288-307	GCTCATCTTCCGGCTTGAGT	
Jhamt	AB113578.1	qPCR	JHAMT_qPCR_FOR	478-497	CCAGGAACGAGCTTTCCGGA	108.2%
			JHAMT_qPCR_FOR	638-662	CGGGATCTTCATTGTCATGGTAAGG	
lebocin	NM_001044003.1	qPCR	LEB_qPCR_FOR	73-93	CCAGAGGTTCATCCAGCCGAC	105%
			LEB_qPCR_REV	138-157	CGGTTCCTGGCCAGCTTGTC	
		Polymorphisms	LEB_FOR	9-35	TCAACATGTACAAGTTTTTAGTATTCA	
			LEB_REV	764-786	GAGAGAAATTTGTTGCAAAGCAA	
moricin	NM_001043364.2	qPCR	MOR_qPCR_FOR	75-96	CATGTAGTACAGCCGCTCCAGC	98.03%
			MOR_qPCR_REV	161-182	CGTTGGCTGTACTGGCGATATT	
		Polymorphisms	MOR_FOR	55-75	TGTGGCAATGTCTCTGGTGTC	
			MOR_REV	329-359	GAACTGACTATAAGTAAGTACTACAAAGGGG	
Nos	NM_001043498.1	qPCR	NOS_qPCR_FOR	1766-1785	CTCTCCAAGCGCATCAAAGC	96.4%
			NOS_qPCR_REV	1869-1888	CGACATGCAATGTACCTGCG	

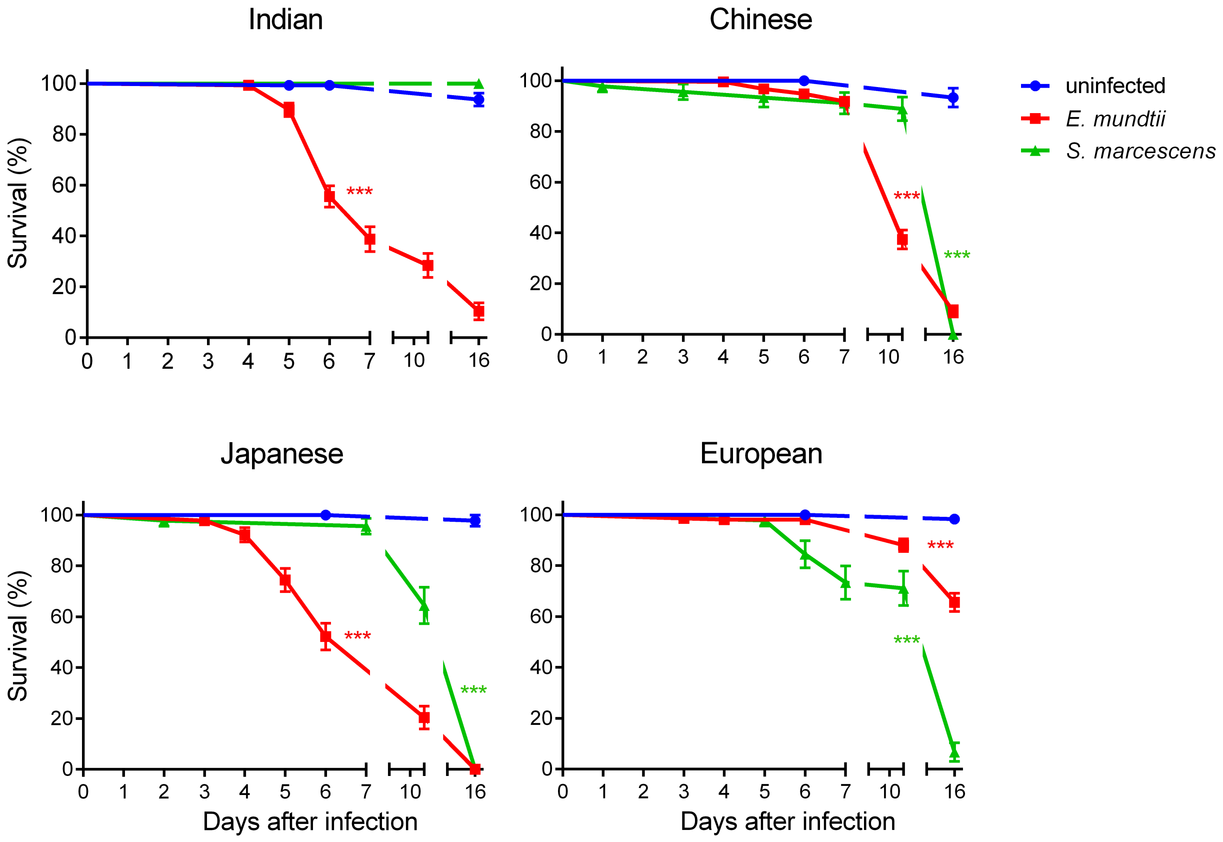

Figure S1. Survival curves (mean %s ± SEM) of the four *B. mori* strains reared in germ-free conditions and orally infected with *E. mundtii* and *S. marcescens*. *** indicates $p< 0.0001$ in the Mantel-Cox test.

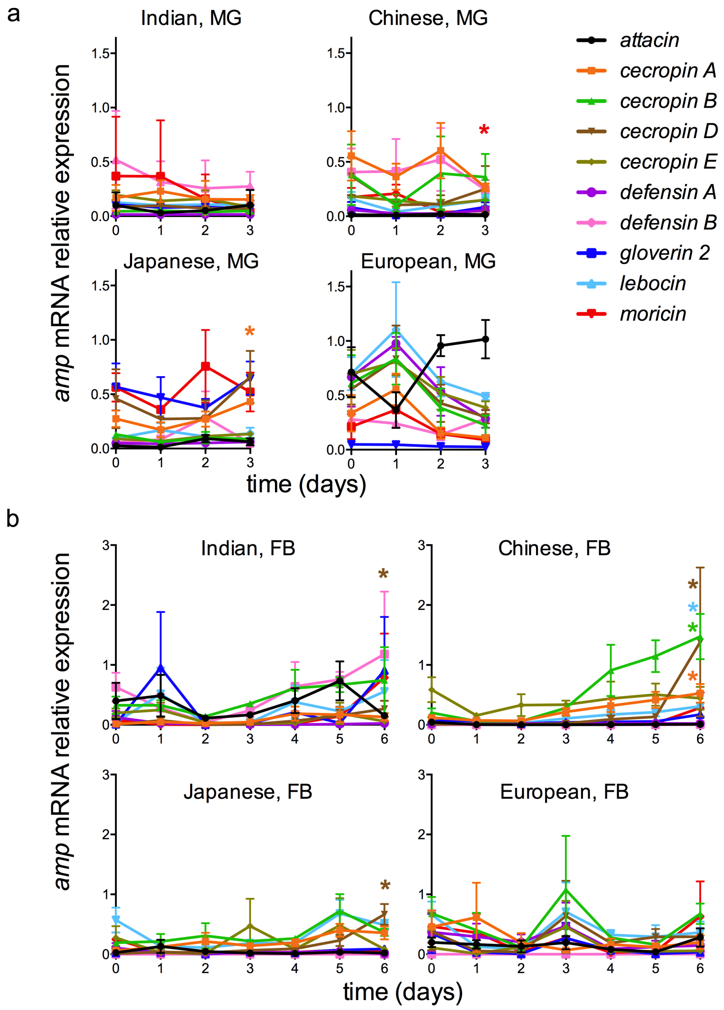

c

AMP gene	Tissue	AMP mRNA relative expression at the moment of the infection (mean ± SEM)				Kruskal-Wallis test			Dunn's *post-hoc* test
		Indian (I)	Chinese (C)	Japanese (J)	European (E)	value	p	S	
attacin	MG	0.10 ± 0.07	0.01 ± 0.006	0.02 ± 0.01	0.71 ± 0.22	8.23	0.01	s	E > C p<0.05;the other comparisons p>0.05, ns
	FB	0.39 ± 0.30	0.04 ± 0.03	0.02 ± 0.01	0.20 ± 0.07	5.82	0.11	ns	
cecropin A	MG	0.16 ± 0. 07	0.55 ± 0.22	0.27 ± 0.07	0.33 ± 0.17	2.89	0.45	ns	
	FB	0.02 ± 0.01	0.12 ± 0.03	0.06 ± 0.03	0. 45 ± 0.27	8.74	0.006	s	E > I p<0.05;the other comparisons p>0.05, ns
cecropin B	MG	0.04 ± 0.02	0.38 ± 0.28	0.13 ± 0.03	0.61 ± 0.25	7.30	0.003	s	E > I p<0.05;the other comparisons p>0.05, ns
	FB	0.33 ± 0.15	0.20 ± 0.07	0.19 ± 0.03	0.68 ± 0.27	1.76	0.67	ns	
cecropin D	MG	0.08 ± 0.01	0.37 ± 0.17	0.46 ± 0.27	0.56 ± 0.14	6.89	0.051	ns	
	FB	0.05 ± 0.005	0.1 ± 0.1	0.1 ± 0.02	0.07 ± 0.03	6.19	0.08	ns	
cecropin E	MG	0.19 ± 0.02	0.18 ± 0.02	0.09 ± 0.02	0.70 ± 0.2	8.74	0.006	s	E > J p<0.05; the other comparisons p>0.05, ns
	FB	0.20 ± 0.07	0.59 ± 0.02	0.27 ± 0.19	0.11 ± 0.03	4.84	0.19	ns	
defensin A	MG	0.01 ± 0.005	0.06 ± 0.02	0.05 ± 0.01	0.67 ± 0.27	8.69	0.007	s	E > I p<0.05; the other comparisons p>0.05, ns
	FB	0.12 ± 0.1	0.01 ± 0.002	0.1 ± 0.07	0.37 ± 0.31	4.23	0.26	ns	
defensin B	MG	0.52± 0.26	0.40 ± 0.21	0.05 ± 0.01	0.28 ± 0.11	6.12	0.08	ns	
	FB	0.62 ± 0.24	0.002 ± 0.001	$6.6*10^{-6} \pm 3*10^{-6}$	0.002 ± 0.0003	9.39	0.02	s	I > J p<0.05; the other comparisons p>0.05, ns
gloverin 2	MG	0.11 ± 0.02	0.08 ± 0.05	0.56 ± 0.21	0.05 ± 0.01	7.66	0.02	s	J > E p<0.05; the other comparisons p>0.05, ns
	FB	0.01 ± 0.005	0.06 ± 0.01	0.02 ± 0.001	0.34 ± 0.32	5.36	0.15	ns	
lebocin	MG	0.12 ± 0.01	0.16 ± 0.06	0.09 ± 0.01	0.69 ± 0.15	7.20	0.04	s	E > J p<0.05; the other comparisons p>0.05, ns
	FB	0.04 ± 0.04	0.07 ± 0.04	0.57± 0.21	0.66 ± 0.22	8.64	0.008	s	J or E > I or C p<0.05
moricin	MG	0.37 ± 0.32	0.17 ± 0.08	0.56 ± 0.13	0.21 ± 0.10	3.31	0.39	ns	
	FB	0.07 ± 0.03	0.02 ± 0.05	0.25 ± 0.12	0.46 ± 0.27	8.89	0.004	s	E > C p<0.05;the other comparisons p>0.05, ns

Figure S2. Local and systemic relative expression of AMP genes in fifth instar silkworms of the four *B. mori* strains reared in germ-free conditions. (**a**, **b**) AMP mRNA relative expression (mean ± SEM, nine individuals pooled in three replicates) in the midgut (**a**, MG) for three days and in fat bodies (**b**, FB) for six days of the fifth larval stage. Coloured *: indicates the specific AMP gene showing significant variations in expression levels during larval development within each strain (Kruskal-Wallis and Dunn's *post-hoc* tests; $p<0.05$). (**c**) Comparisons of AMP expression levels (mean ± SEM) among the four strains just before infection (day 0; S: significance with "s" significant and "ns" not significant).

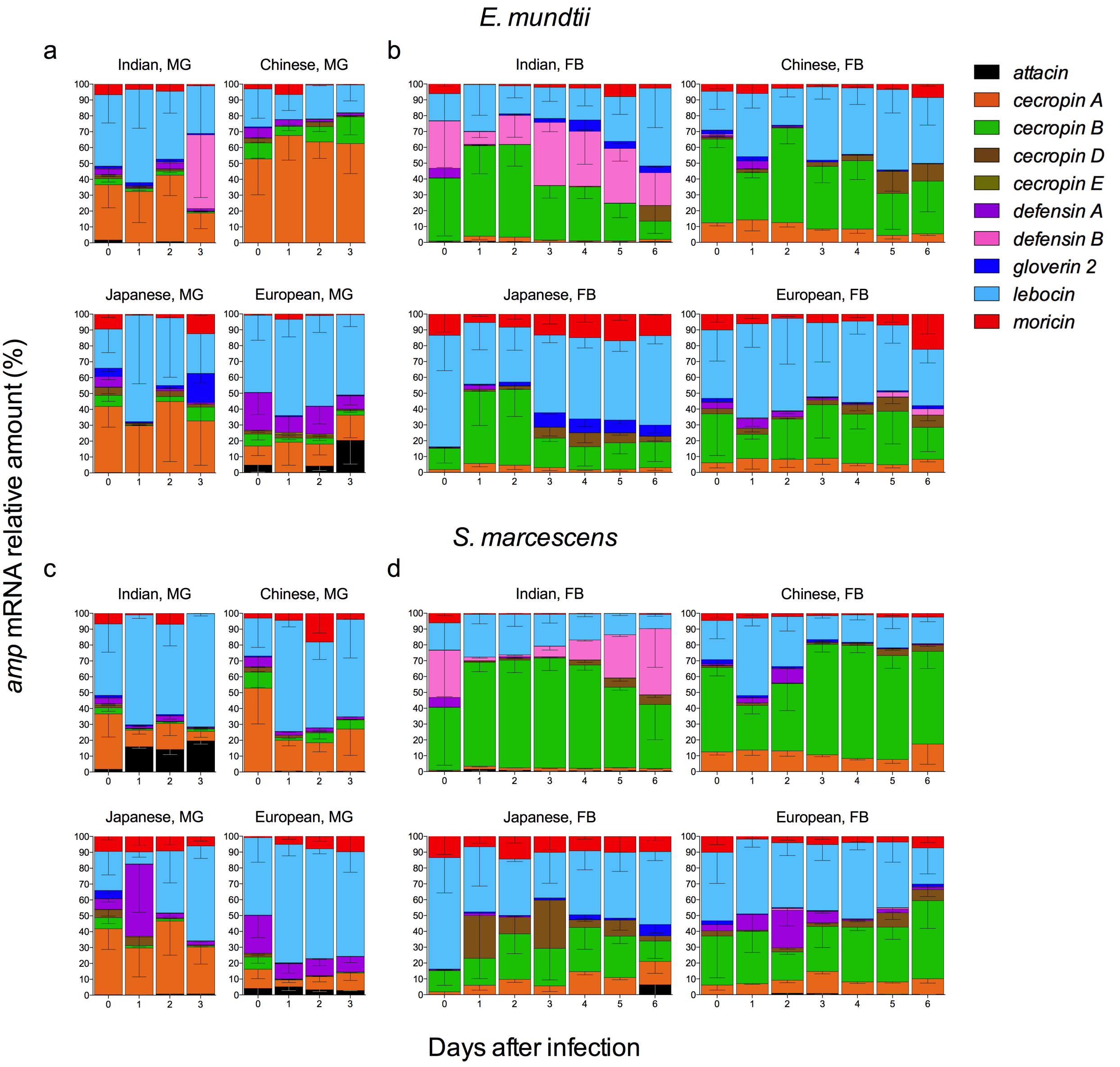

Figure S3. Relative amounts (mean %s ± SEM) of AMP mRNAs in the four *B. mori* strains after *E. mundtii* (a, b) or *S. marcescens* (c, d) infection. Expression values (%) of each AMP gene are normalised to those of *attacin* within the same strain and day of infection. MG: midgut; FB: fat body.

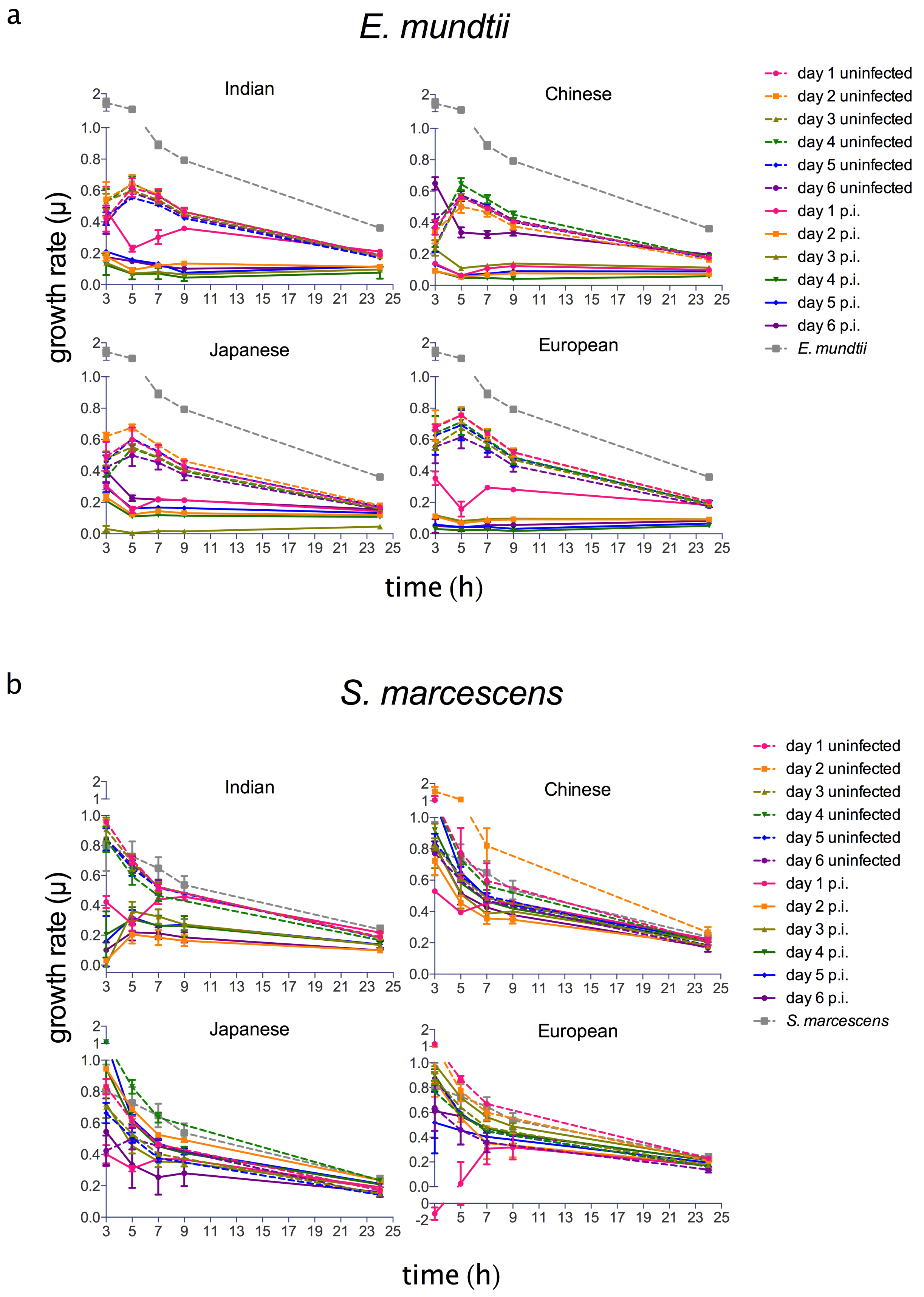

Figure S4. *In vitro* growth rates of *E. mundtii* and *S. marcescens* in the presence of infected and uninfected plasma. *In vitro E. mundtii* (**a**) or *S. marcescens* (**b**) growth rates (µ) measured for 24 h after addition of infected and uninfected plasma collected for six days after bacterial challenge (mean ± SEM; nine plasma in three replicates per time per condition); p.i.: post-infection.

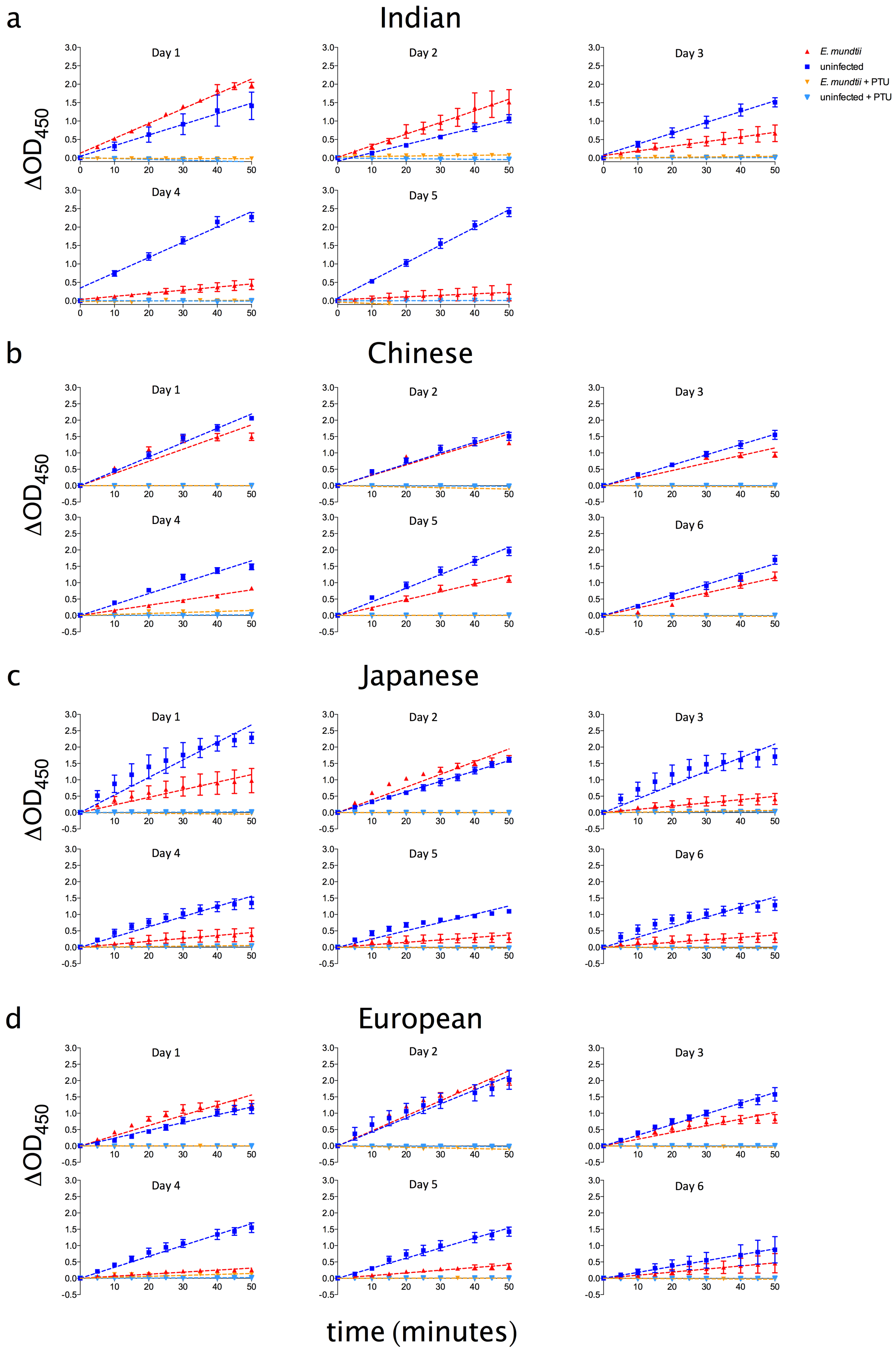

Figure S5. Melanization rates and corresponding regression lines of *E. mundtii* infected and uninfected plasma. Hemolymph was collected from both infected and uninfected silkworms of the Indian (**a**), Chinese (**b**), Japanese (**c**) and European (**d**) strains for six days after bacterial challenge (five days for the Indian infected line). The ΔOD_{450} of infected (red triangles) and uninfected (blue squares) plasma was determined for each 10 min-reading (mean ± SEM; nine plasma in three replicates per day per condition) and regression lines were drawn (dotted lines). As negative control of melanization 2.5 mM phenylthiourea (PTU) was added to both uninfected (light blue) and infected (yellow) plasma.

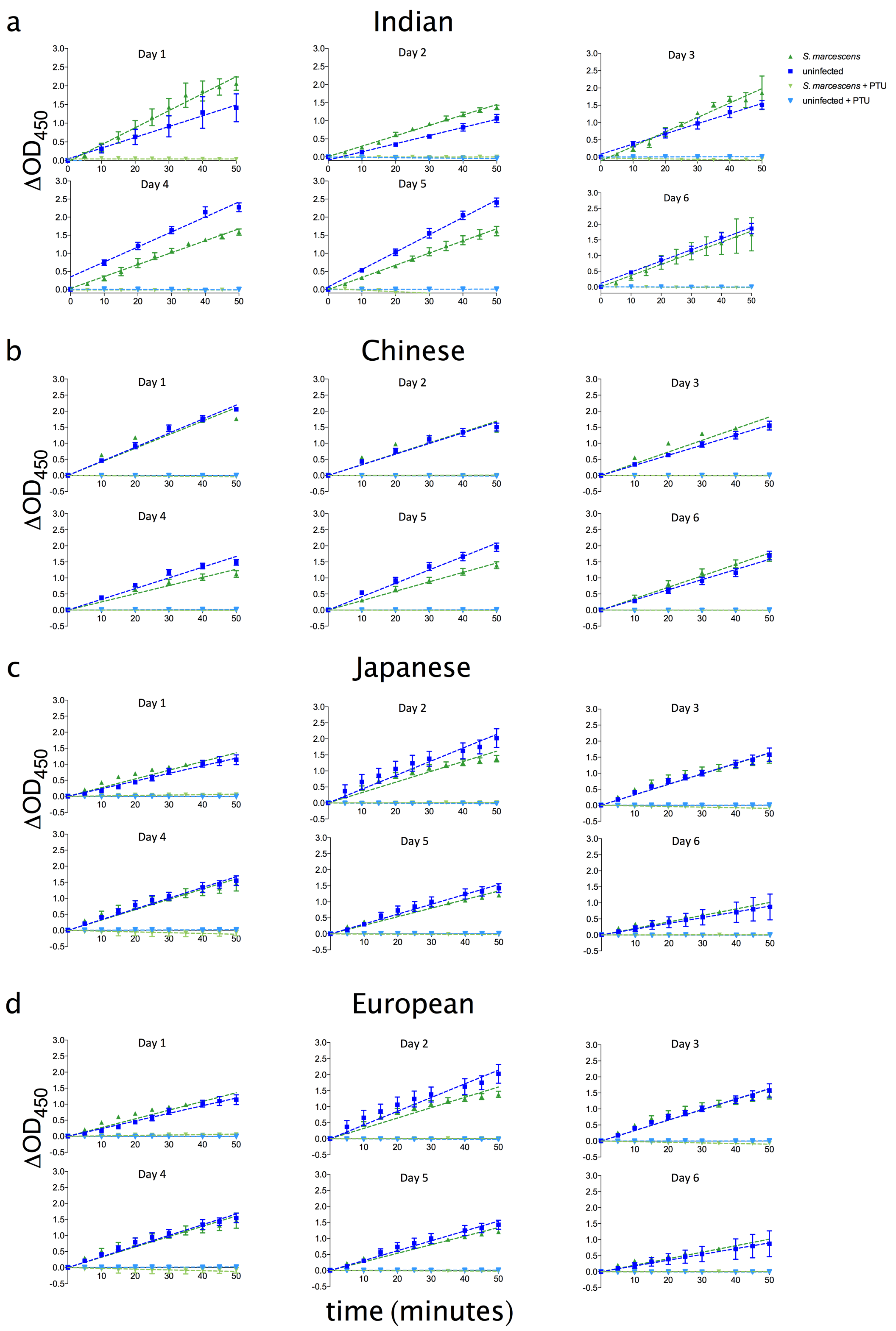

Figure S6. Melanization rates and corresponding regression lines of *S. marcescens* infected and uninfected plasma. Hemolymph was collected from both infected and uninfected silkworms of the Indian (**a**), Chinese (**b**), Japanese (**c**) and European (**d**) strains for six days after bacterial challenge. The ΔOD_{450} of infected (green triangles) and uninfected (blue squares) plasma was determined for each 10 min-reading (mean ± SEM; nine plasma in three replicates per day per condition) and regression lines were drawn (dotted lines). As negative control of melanization 2.5 mM phenylthiourea (PTU) was added to both uninfected (light blue) and infected (light green) plasma.
